# *PARP3* Promotes AML Progression via Activation of PI3K/AKT/mTOR Signaling

**DOI:** 10.3390/cancers17183076

**Published:** 2025-09-20

**Authors:** Tingyong Cao, Yurong Zhang, Huan Liu, Hongbin Zhang, Liangliang Li, Xiaoli Li, Li Zhao

**Affiliations:** 1 The First Clinical Medical College, Lanzhou University, Lanzhou 730000, China; 120220902760@lzu.edu.cn; 2 Department of Hematology, The Second Hospital & Clinical Medical School, Lanzhou University, Lanzhou 730000, China; 3 Department of Nephrology, Gansu Provincial Hospital, Lanzhou 730000, China; 4 Gansu Provincial Clinical Medical Research Center for Molecular Diagnosis and Treatment of Hematological Diseases, The First Hospital, Lanzhou University, Lanzhou 730000, China; 5 The Laboratory Center, The First Hospital, Lanzhou University, Lanzhou 730000, China

**Keywords:** AML, *PARP3*, PI3K/AKT/mTOR signaling, apoptosis, cell cycle, migration

## Abstract

**Simple Summary:**

Acute myeloid leukemia (AML) remains an incurable malignant tumor that constitutes a major threat to human health. Refractory and relapsed cases remain two major causes of treatment failure. Therefore, there is an urgent need for new diagnostic and prognostic biomarkers to provide more options for AML treatment. The role of *PARP3* in AML has not been previously investigated. We analyzed the pattern of *PARP3* expression through GEO databases and data from our center and concluded that *PARP3* was significantly overexpressed and correlated with adverse clinical outcomes in AML. Furthermore, functional assays demonstrated that *PARP3* drives AML progression by stimulating proliferation and migration through the PI3K/AKT/mTOR axis, identifying it as a promising therapeutic target.

**Abstract:**

**Background**: Acute myeloid leukemia (AML) remains a hematopoietic clonal malignancy that is characterized by a poor prognosis, largely attributable to chemotherapy resistance and a high incidence of post-chemotherapy relapse. Therefore, the identification of novel molecular markers is crucial for optimizing treatment regimens and improving outcomes for this disease. **Methods**: We first investigated the expression levels of poly(ADP-ribose)polymerase 3(*PARP3*) mRNA in data from our center and the Gene Expression Omnibus (GEO), then explored the role of *PARP3* in AML through cell experiments. **Results**: Our results demonstrated that the expression levels of *PARP3* were significantly elevated in AML samples compared to controls (*p* < 0.05). Based on the median expression of *PARP3*, 151 cases of AML from TCGA data were divided into two groups. The results showed that *PARP3*-high group had markedly shorter overall survival (OS) than the *PARP3*-low group (OS: median: 1.18 vs. 3.88 years; *p* < 0.001). The overexpression of *PARP3* was correlated with older age and high-risk stratification in the AML from TCGA data (*p* < 0.05). Finally, we confirmed that specifically down-regulating *PARP3* expression impaired AML cell proliferation, disrupted cell cycle process, inhibited migration, accelerated apoptosis, and impaired the PI3K/AKT/mTOR signaling pathway in vitro. **Conclusions**: *PARP3*-mediated activation of the PI3K/AKT/mTOR signaling pathway enhances AML cell proliferation and migration, identifying it as a potential therapeutic target for poor-prognosis AML.

## 1. Introduction

Acute myeloid leukemia (AML) is a type of clonal hematopoietic malignancy driven by diverse mutations and cytogenetic alterations marked by significant tumor heterogeneity, which is an underlying factor in the difficulty of treatment. Recent advances in genomic, immunologic, and molecular research have fostered innovation in AML management, leading to enhancements in conventional chemotherapy and the development of novel targeted agents [1]. Notwithstanding these improvements, the prognosis for AML patients continues to be unfavorable, largely owing to chemoresistance and post-treatment relapse [2]. It is critically imperative to elucidate the molecular mechanisms underlying drug resistance and disease recurrence to refine risk stratification and optimize therapeutic strategies.

The poly-ADP-ribose polymerase (PARP) family encompasses 17 genes that encode enzymes performing nuclear protein ADP-ribosylation, a post-translational modification critical for DNA damage response and repair [3]. Recently, the PARP family has become recognized as a crucial regulator in cancer biology. Multiple PARP members have been implicated in the progression of hematologic malignancies, including AML, diffuse large B-cell lymphoma, and multiple myeloma [4,5,6]. In the AML, studies have revealed that PARP1 is overexpressed in the AML with FMS-related receptor tyrosine kinase 3 internal tandem duplications (FLT3-ITD) mutation, driving PARylation of STAT5 to sustain partial STAT5 signaling activation and promote AML survival and proliferation [7]. Some studies revealed that both PARP14 and PARP10 are overexpressed in the AML and predict a shorter overall survival [8,9]. Mechanistically, PARP10 could be involved in transcription and epigenomic regulation in AML, PARP14 promotes the proliferation of AML by excessively activating the NF-κB signaling pathway, further upregulating HIF-1α expression and thereby augmenting glycolytic flux in AML cells. Several studies have confirmed a preclinical activity for the most-studied PARP inhibitors (PARPi)—olaparib, niraparib, and talazoparib—in combination with cytotoxic, hypomethylating, and histone deacetylase inhibitors or targeted drugs in AML [10,11,12,13]. These data indicate that different PARP subtype molecules may play distinct roles in AML and that specifically targeting PARP molecules may have significant value.

The main functions of *PARP3* have been identified as programmed double-strand break repair, stress-induced break repair, chromosome rearrangement, and mitotic segregation [14,15,16,17]. In recent years, *PARP3* also has been reported to be associated with solid tumor cell hyperproliferation, cell survival, and Cytoskeleton-dependent processes [18]. Some studies have indicated that *PARP3* is overexpressed in aggressive breast cancer and primary glioblastoma [19,20]. Inhibition of *PARP3* expression can sensitize metastatic breast cancer to the chemotherapeutic agent vinorelbine [21]. Silencing *PARP3* expression improves the therapeutic efficacy of radiotherapy in glioblastoma [22]. Recent studies also demonstrate that *PARP3* enhances cisplatin sensitivity by modulating the PDGF and G-coupled signaling pathways [23]. Collectively, these results imply that *PARP3* may represent a potential novel target for overcoming various malignant tumors. However, the role of *PARP3* has not yet been studied in the AML. Therefore, we hypothesize that targeting *PARP3* may achieve a potential anti-leukemia effect by disrupting genomic stability and cell growth.

Therefore, this study investigated the expression levels of *PARP3* mRNA in AML patients and normal samples from the TCGA and GEO databases, analyzed the correlations between *PARP3* expression levels and clinical characteristics, and elucidated the prognostic value of *PARP3* expression in AML. Then, we collected 23 new AML patients to validate the results of the analysis of public data. Furthermore, we performed cell experiments in vitro to analyze the cellular function of *PARP3* and further elucidate the molecular significance of *PARP3*.

## 2. Materials and Methods

### 2.1. Gene Expression Profiling Data Sets, Patients and Controls

We obtained RNA expression, clinical and laboratory parameters, gene mutation, and survival data for 151 newly diagnosed AML patients from The Cancer Genome Atlas (TCGA-AML) cohort via the TCGA portal (https://portal.gdc.cancer.gov/, accessed on 17 January 2024). GSE13159, GSE15061, and GSE14468 were retrieved from the Gene Expression Omnibus (GEO) database (https://www.ncbi.nlm.nib.gov/geo/, accessed on 17 January 2024). The *PARP3* expression differences between AML patients and normal samples, as well as among favorable-, intermediate-, and poor-risk stratification groups, were analyzed using three datasets (GSE13159, GSE15061, and GSE14468). Further, 23 newly diagnosed AML patients before treatment and 11 normal individuals were recruited from the Second Hospital of Lanzhou University (Supplementary Table S1). Written informed consent was obtained from all participants (patients and healthy samples), and the study was conducted in accordance with the ethical standards of the Second Hospital of Lanzhou University, which granted formal approval for the protocol. AML patients included in this study were required to meet the diagnostic criteria outlined by both the French–American–British (FAB) classification and the World Health Organization (WHO) [24,25] standards and had an unequivocal diagnosis that was established through the integration of clinical, morphological, immunophenotypic, and genetic characteristics. Then, the relative expression of *PARP3* mRNA was tested through Real-time Polymerase Chain Reaction (qPCR) on fresh bone marrow (BM) mononuclear cells within 8 h of sample collection.

### 2.2. RNA Extraction and RT-qPCR

Total RNA was extracted from BM mononuclear cells or cell lines with RNAiso Plus (Takara, Beijing, China). According to the manufacturer’s instructions, first-strand cDNA was synthesized from total RNA by reverse transcription using the PrimeScript™ RT reagent Kit with gDNA Eraser (Code. No. RR047A. TaKaRa, Beijing, China). The mRNA level was quantified using the TB Green Premix Ex Taq II (Tli RNaseH Plus) kit (Code No. RR820A.TaKaRa, Beijing, China). Relative mRNA expression change was determined using the 2^−ΔΔCt^ method and *β-actin* served as the endogenous normalization control. All primer sequences are provided in Table 1.

### 2.3. Cell Culture and RNA Interference

The AML cell line MOLM-13 and THP-1 cells were all acquired from the Central Laboratory of the First Hospital of Lanzhou University. All cells were maintained in RPMI 1640 (Meilunbio, Dalian, China) medium containing 10% fetal bovine serum (Excell Fetal Bovine Serum Prime, Uruguay) and 1% penicillin–streptomycin (solarbio, Beijing, China). The cells were maintained in a humidified, sterile incubator at 37 °C with 5% CO_2_. Lentiviruses for *PARP3* knockdown (shRNA), overexpression constructs, and empty vectors were sourced from Genechem (Shanghai, China), the infection of MOLM13 and THP-1 cells was performed at an appropriate multiplicity of infection (MOI) following the manufacturer’s instructions. Successfully transduced cells were then selected with puromycin (0.4–2 μg/mL, Beyotime, Shanghai, China). Target sequences for shRNA used in this study included

*PARP3* shRNA1: GCACCATATCAACACGGACAA;
*PARP3* shRNA2: GCACCTGAGTACAAGGTGATA;
*PARP3* shRNA2: CCAGTCAAAGATCAACCACTT.

The construct for *PARP3* overexpression has the following element sequence: Ubc-MCS-3FLAG-CBh-gcGFP-IRES-puromycin. The vector is designated as GV492.

### 2.4. Cell Proliferation Assay

Cell proliferation was quantified at 24, 48, 72, and 96 h using a CCK-8 assay (sparkjade, ShanDong, China) according to the manufacturer’s protocol. Cells were seeded at 1.5 × 10^4^ cells/well, and OD450 values were measured after 2 h of incubation with reagent addition using a Thermo Multimode microplate reader.

### 2.5. Colony Formation Experiment

Cells were plated at a density of 1 × 10^3^ cells per well in 12-well plates and maintained in culture medium supplemented with 20% fetal bovine serum (FBS) and 1.6% methylcellulose. After two weeks, the number of visible colonies was counted and compared (an overall image for each well was captured using a phone camera).

### 2.6. Flow Cytometry Analysis

To assess cell cycle distribution, stable shPARP3 transfectants, overexpression *PARP3* transfectants, and wildtype control cells were collected, washed with phosphate-buffered saline (PBS), and subjected to staining. According to the manufacturer’s (MultiSciences Biotech, Hangzhou, China) protocol, cells were incubated for 30 min at room temperature in the dark using a binding buffer solution containing 1 mL of DNA staining solution and 10 µL of permeabilization solution. The stained cells were then analyzed on a FACS Calibur flow cytometer (BD Biosciences, San Jose, CA, USA), and the resulting data were processed using FlowJo software (v10.8.1, TreeStar, Woodburn, OR, USA) to determine cell cycle phase. For apoptosis analysis, harvested cells were washed with PBS and resuspended in 515 µL of a staining mixture consisting of 5 µL Annexin V-FITC (or Annexin V-PE), 10 µL of 7-aminoactinomycin D (7-AAD), and 500 µL of 1× binding buffer (MultiSciences Biotech, Hangzhou, China). A FACS Calibur (BD Biosciences, San Jose, CA, USA) was used to examine the percentage of apoptotic cells. Each test was performed in triplicate.

### 2.7. Transwell Assay

Cell migration was measured using the Transwell assay. Cells (THP-1 and MOLM13) that had been cultured for 24 h in serum-free medium were seeded in the upper chamber (200 µL, 15 × 10^4^ cells/chamber), meanwhile the lower chambers were filled with 600 µL RPMI medium (Meiluncell, Jinhua, China) supplemented with 20% FBS (Excell Fetal Bovine Serum Prime, Uruguay). Following incubation for 24 h at 37 °C, non-migratory cells on the upper surface of the upper chamber were removed using a cotton applicator. The migratory cells attached to the lower membrane of the upper chamber were then fixed with 4% paraformaldehyde for 20 min and subsequently stained with 0.1% crystal violet (Biosharp, Hefei, China) for 20 min. Finally, the membrane was gently rinsed with PBS to remove excess stain. The results were examined and imaged using an Olympus microscope (CKX41, Olympus Corporation, Shinjuku, Japan).

### 2.8. Western Blot

Cells were lysed by incubation on ice for 30 min in RIPA buffer [1× PMSF (Solarbio, China), 1× protease inhibitor cocktail (Epizyme, Shanghai, China)]. After centrifugation at 12,000× *g* rpm at 4 °C for 15 min, cleared suspension was quantified using a BCA protein assay kit (Solarbio, Beijing, China). Cellular lysis was performed by incubation in RIPA buffer [supplemented with 1× PMSF (Solarbio, Beijing, China) and a 1× protease inhibitor cocktail (Epizyme, Shanghai, China)] on ice for 30 min. After centrifugation (12,000× *g*, 15 min, 4 °C), the protein concentration in the supernatant was measured by employing a bicinchoninic acid (BCA) assay kit (Solarbio, Beijing, China). SDS—PAGE was employed to segregate similar protein volumes (30 μg) following transfer to polyvinylidene fluoride (PVDF) membranes (Millipore, IPVH00010, Billerica, MA, USA). The membranes were immunoblotted with primary antibodies that target *PARP3* (1:1000, Proteintech, Wuhan, China), p—mTOR, mTOR, p—AKT, AKT (1:1000, Affinity biosciences, Changzhou, China), P-PI3K, PI3K (1; 1000, Ab-mart, Shanghai, China), E-cadherin, N-cadherin, TWIST1, Snail (1:1000, Cell Signaling Technology, Danvers, MA, USA), BCL2, BAX, Vimentin (1:1000, Wanleibio, Shenyang, China), and *β-actin* (1:50,000, HuaBio, Hangzhou, China) overnight at 4 °C after a blocking step using 5% non-fat milk. Subsequent to incubation with secondary antibodies (1:10,000, Epizyme, Shanghai, China), the blots were quantified with an ECL kit (Meiluncell, Jinhua, China) and then scanned with an Amersham Imager 680 (Cytiva, Marlborough, MA, USA) and/or an SH—523 chemiluminescence imaging system (Hangzhou Shenhua Technology, Hangzhou, China). For the expression of different proteins in the same blots, partly blotted membranes were incubated with fast stripping buffer (meilunbio) followed by TBST washes and treated as mentioned above. Band intensities from western blots were quantified using ImageJ 1.54f software, with *β-actin* serving as the internal loading control.

### 2.9. Statistical Analysis

All statistical analyses were conducted using SPSS 27.0 (SPSS software, Chicago, IL, USA) or Prism GraphPad 9.0 (GraphPad Software, Boston, MA, USA) and expressed as percentages or mean ± standard deviation (SD). The Spearman χ^2^ test was applied to analyze the correlation between *PARP3* and clinical and laboratory characteristics. Overall survival (OS) was estimated using the Kaplan–Meier method. Univariate and multivariate Cox proportional models were constructed to identify independent prognostic factors influencing OS. An unpaired Student’s *t*-test was used to compare the means between two groups, and One-way ANOVA was applied to assess the differences among three or more groups, respectively. Dunnett’s multiple comparisons test or Tukey’s multiple comparisons test were used for the post hoc test following ANOVA. All statistical tests were 2-sided; *p* values < 0.05 were defined as statistically significant.

## 3. Results

### 3.1. PARP3 Is Overexpressed in AML and Correlates with Poorer Survival

**Figure 1:**
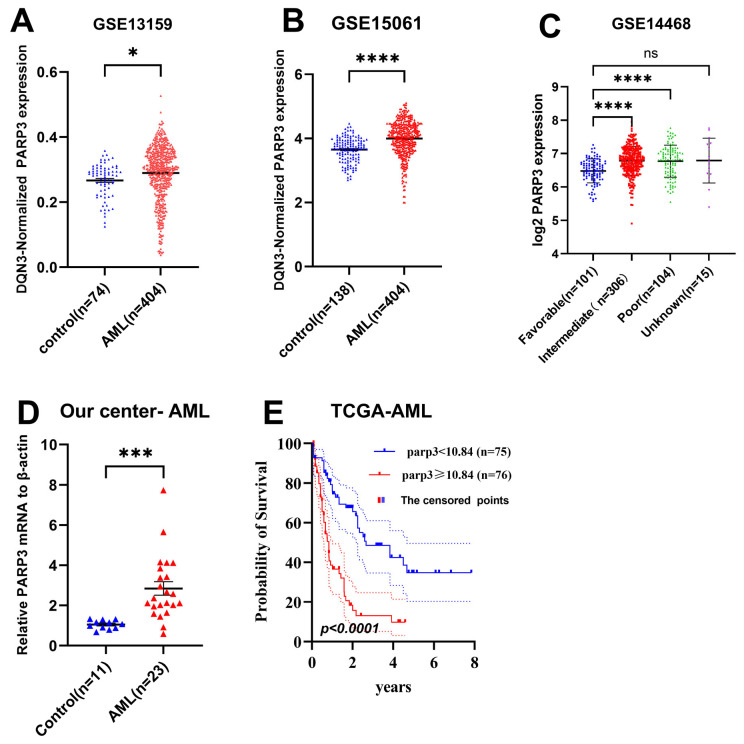
Differential expression of *PARP3* between AML samples and normal controls in GSE13159, GSE15061, GSE14468, and clinical datasets. (**A**) GSE13159: AML n = 541, Control n = 74; (**B**) GSE15061: AML n = 404, Control n = 138; (**C**) GSE14468: good risk AML = 101, intermediate risk AML = 306, poor risk AML = 104, no classified AML = 15; (**D**) Our center data: AML = 23, Control n = 11. (**E**) Differential overall survival difference of AML patients with high-*PARP3* group versus low-*PARP3* group in TCGA. An unpaired *t* test was performed to evaluate the statistical significance of the observed expression differences. * *p* < 0.05; *** *p* < 0.001; **** *p* < 0.0001; ns: Non-Significant.

To investigate the transcriptomic profile of the *PARP3* gene in AML, we first assessed the mRNA levels of *PARP3* in the GSE13159, GSE15061, and GSE14468 GEO databases. Our findings revealed a marked difference in *PARP3* expression between AML samples and normal bone marrow controls (*p* < 0.05, Figure 1A,B), with markedly elevated *PARP3* levels observed in intermediate- and high-risk AML subgroups (*p* < 0.05, Figure 1C). Next, we performed a validation analysis by comparing 23 newly diagnosed AML patients and 11 control subjects with non-hematologic malignancies recruited from our center, of which 12 were men and 11 were women. Our findings revealed a similar differential expression pattern, where in comparison to normal bone marrow controls, *PARP3* expression was significantly higher in AML patients (*p* < 0.05, Figure 1D). To explore a deeper understanding of the significance of *PARP3* in the clinical outcome of AML patients, we plotted the OS Kaplan–Meier curves for AML patients between the high- and low-*PARP3* groups (cutoff: *PARP3* median expression) using the TCGA-AML data. The results indicated that elevated *PARP3* levels were significantly associated with inferior OS (OS: median: high-*PARP3* group 1.18 vs. low-*PARP3* group 3.88 years; *p* < 0.001; Figure 1E). Therefore, *PARP3* might participate in the progression of AML.

### 3.2. Association Between PARP3 Expression Levels and Clinical/Laboratory Characteristics in AML Patients

We next examined the interaction between *PARP3* expression levels and clinicopathological features in AML samples from the TCGA database (Table 2). The results showed that elevated *PARP3* expression was significantly linked to advanced age (*p* = 0.001), higher ELN risk categories (*p* = 0.011), and unfavorable cytogenetic risk profiles (*p* = 0.017). Based on the ELN risk stratification, patients in the intermediate- and poor-risk groups demonstrated markedly higher *PARP3* expression, whereas those classified as having favorable risk showed lower expression levels (*p* = 0.001).

A similar pattern was observed in the cytogenetic risk classification (*p* = 0.001). No notable differences were observed in gender, white blood cell count, bone marrow blast count, peripheral blood blast count, gene mutations, or FAB classification between the high- and low-*PARP3* expression groups (*p* > 0.05) (Table 2). Collectively, these findings indicate that elevated *PARP3* expression is a hallmark of high-risk AML and serves as a biomarker predictive of adverse clinical outcomes.

### 3.3. Cox Regression Analyses Indicated PARP3 as an Independent Factor for AML Prognosis

The prognostic significance of the following categorical variables was assessed: *PARP3* expression level (high vs. low), age (<60 vs. ≥60 years), WBC count (<50 vs. ≥50 × 10^9^/L), sex (male vs. female), mutation status of six common genes (NPM1, FLT3, IDH1, IDH2, NRAS, KRAS; mutant vs. wildtype), and ELN 2017 risk classification (favorable vs. intermediate vs. poor). Factors showing at least borderline significance (*p* < 0.1) in univariate Cox proportional hazards analyses for overall survival (OS) were included in subsequent multivariate analysis. Among TCGA AML patients, elevated *PARP3* expression was significantly correlated with worse OS in univariate analysis (HR = 3.190, 95% CI 2.004–4.979; *p* = 0.000, Table 3). Older age, higher WBC count, and ELN risk were significant predictive factors resulting in a reduced OS (*p* < 0.05, respectively, Table 3), while the other clinical and molecular factors had no effect on OS (all *p* > 0.05). Multivariate Cox regression analysis revealed that high PAPR3 expression was an independent risk factor for the OS of AML patients (HR = 1.035, 95% CI 1.011–1.059; *p* = 0.004), even in the presence of the other covariates. Older age, higher WBC count, and intermediate/adverse ELN risk were also correlated with an inferior OS in TCGA AML patients (*p* < 0.05, respectively, Table 3).

### 3.4. PARP3 Knockdown Impaired AML Cell Proliferation, Induced Cell Apoptosis, and Destroyed the Cell Cycle

**Figure 2:**
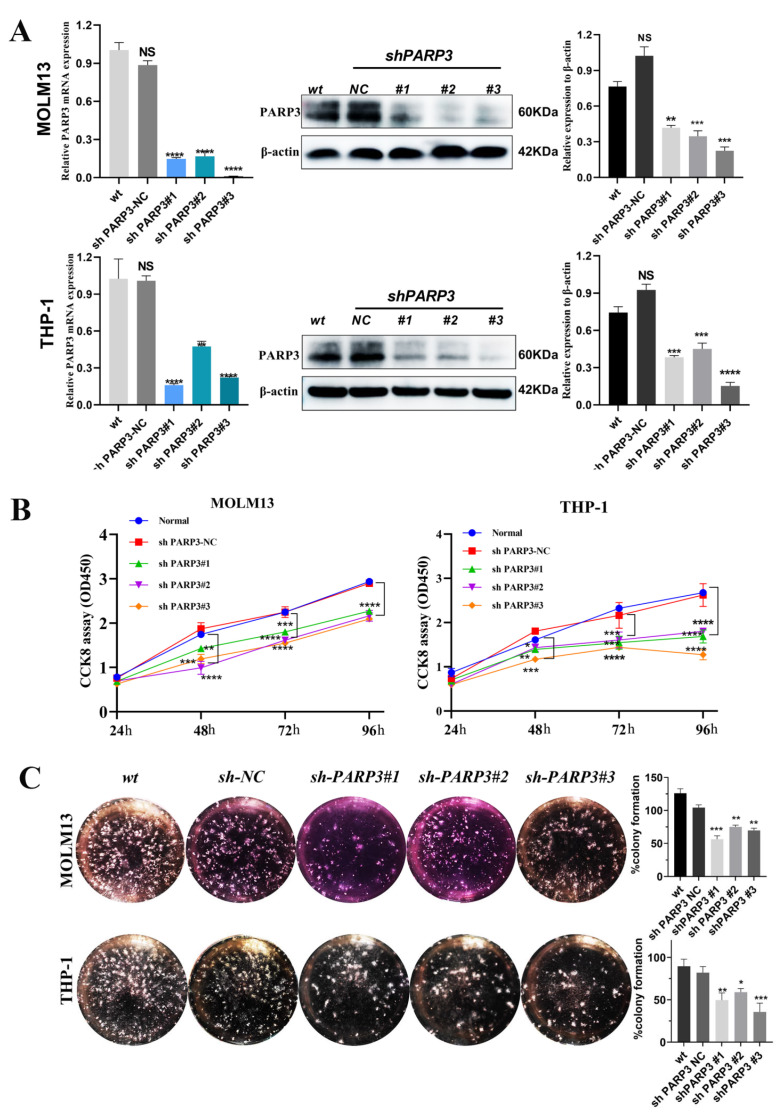
*PARP3* promotes AML cell expansion in vitro. (**A**) Efficacy of *PARP3* knockdown in THP-1 and MOLM13 cells via lentivirus transfection was measured using RT-PCR and WB. (**B**,**C**) *PARP3* knockdown suppresses AML cell proliferation, as measured by CCK-8 and colony formation assays. Data are presented as mean ± SD of three independent experiments. NS, not statistically significant, * *p* < 0.05, ** *p* < 0.01, *** *p* < 0.001, and **** *p* < 0.0001 vs. the sh-*PARP3*-NC group. AML, acute myeloid leukemia; CCK, cell counting kit; SD, standard deviation; wt, wildtype—no lentiviruses transfection; NC, negative control—empty vector of lentiviruses transfection; sh: short hairpin RNA.

To investigate the biological function of *PARP3* in AML cells, the lentiviruses for *PARP3* shRNA and the empty vector transfected into the MOLM13 and THP-1 cells and the knockdown efficiency was examined using RT-qPCR and WB (Figure 2A; the original western blots images are shown in Supplementary Figure S2).

**Figure 3:**
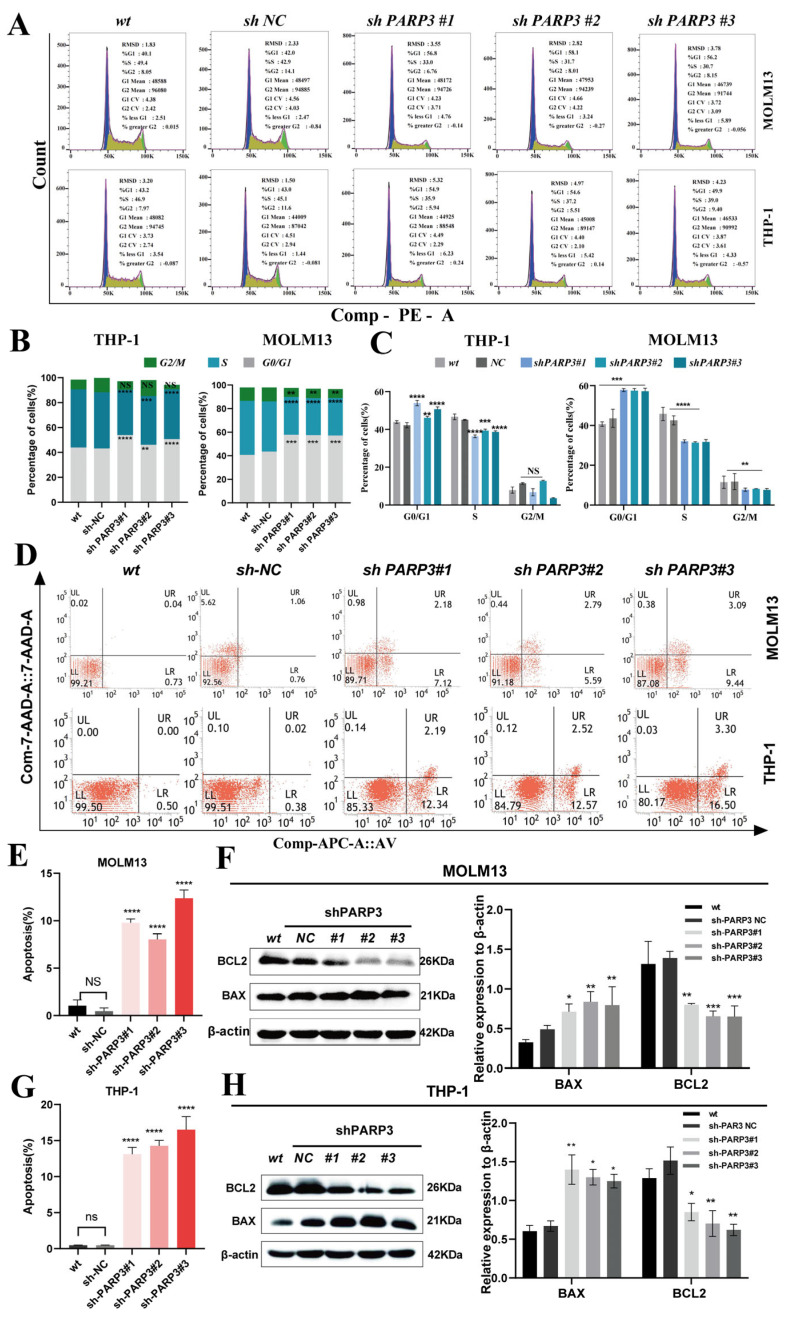
*PARP3* enhances AML cell proliferation in vitro. (**A**–**C**) Effect of *PARP3* knockdown on cell cycle regulation in AML cells. (**D**,**E**,**G**) Effect of *PARP3* knockdown on cell apoptosis regulation in AML cells. (**F**,**H**) Expression of apoptosis-associated proteins BCL2 and BAX in *PARP3*-knockdown and control AML cells. Data are presented as mean ± SD of three independent experiments. NS, not statistically significant, * *p* < 0.05, ** *p* < 0.01, *** *p* < 0.001, and **** *p* < 0.0001 vs. the sh-*PARP3*-NC group. AML, acute myeloid leukemia; SD, standard deviation. wt, wildtype—no lentivirus transfection; NC, negative control—empty vector of lentiviruses transfection; sh: short hairpin RNA.

We found that *PARP3* knockdown inhibited AML cell proliferation using a CCK-8 assay (Figure 2B). To verify this observation, we performed colony formation assays, which yielded corroborating results (Figure 2C). Prior studies have established that *PARP3* plays a crucial role in mitosis [21]. Cell cycle assays revealed that *PARP3* knockdown resulted in an increase in the proportion of MOLM13 and THP-1 cells in the G1/G0 phase of the cell cycle and a concomitant decrease in S phase cells. However, cell cycle assays revealed a decrease in the proportion of cells in the G2/M phase in the MOLM13 cells only following *PARP3* knockdown compared with the control, whereas no significant differences in the proportion of cells in the G2/M phase were seen in THP-1 cells (Figure 3A–C). Furthermore, flow cytometric analysis demonstrated a marked increase in apoptosis upon *PARP3* knockdown in both THP-1 and MOLM13 cell lines compared to control groups (Figure 3D,E,G). To corroborate these findings, we examined key apoptotic regulators by western blotting. *PARP3* knockdown consistently reduced the level of the anti-apoptotic protein BCL2 and enhanced expression of the pro-apoptotic protein BAX in both cell lines, further supporting the flow cytometry results (Figure 3F,H). Additionally, *PARP3* overexpression was also induced in MOLM13 and THP-1 cells by lentivirus transfection. CCK8 assays and flow cytometry analyses were performed to explore the effects of *PARP3* on cell proliferation, the cell cycle, and apoptosis in MOLM13 and THP-1 cells, respectively. The results demonstrated that *PARP3* overexpression enhanced the proliferation of MOLM13 and THP-1 cells; however, no notable differences in apoptosis or cell cycle distribution were observed between *PARP3*-overexpressing and control cells in either cell line (Supplementary Figure S1).

### 3.5. PARP3 Promotes the Migration of AML Cells In Vitro

**Figure 4:**
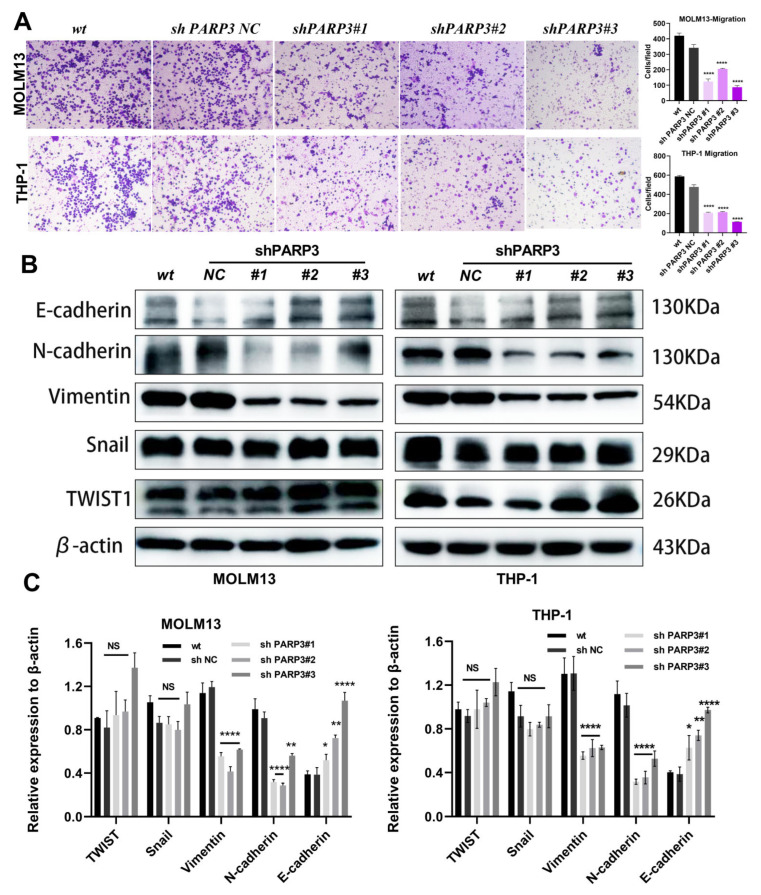
*PARP3* promotes the migration of AML cells in vitro. (**A**) Transwell assays were used, to assess the migration ability of AML cells. Magnification, ×200. (**B**,**C**) Expression of EMT-associated proteins E-cadherin, N-cadherin, Vimentin, Snail, and TWIST1 in *PARP3* knockdown and control AML cells. Data are presented as the mean ± SD of three independent experiments. NS, not statistically significant, * *p* < 0.05, ** *p* < 0.01, and **** *p* < 0.0001 vs. the shPARP3-NC group. AML, acute myeloid leukemia; SD, standard deviation; EMT, epithelial–mesenchymal transition; wt, wildtype—no lentivirus transfection; NC, negative control—empty vector of lentivirus transfection; sh: short hairpin RNA.

Accumulating evidence has indicated a link between extramedullary infiltration and the epithelial–mesenchymal transition (EMT) in AML [26]. Recent studies have further revealed EMT-related modulators as critical regulators in AML biology [27]. Our previous work also demonstrated that the mesenchymal stem cells obtained from AML patients can induce chemoresistance and promote an EMT-like phenotype in AML cells [28]. Therefore, to investigate whether *PARP3* influences AML cell migration, we conducted Transwell assays using THP-1 and MOLM13 cells. The results revealed that *PARP3* knockdown significantly suppressed the migratory ability of both cell lines compared with controls (Figure 4A). We further examined the expression of EMT-related proteins by WB assays. *PARP3* knockdown led to a marked increase in the epithelial marker E-cadherin, whereas the mesenchymal markers N-cadherin and vimentin were downregulated (Figure 4B,C). However, there were no notable differences in the expression of the mesenchymal transcription factors Snail and Twist1. These results verified that *PARP3* promotes the EMT and migration of THP-1 and MOLM13 cells in vitro.

### 3.6. Potential Molecular Mechanism Mediated by PARP3 in AML

**Figure 5:**
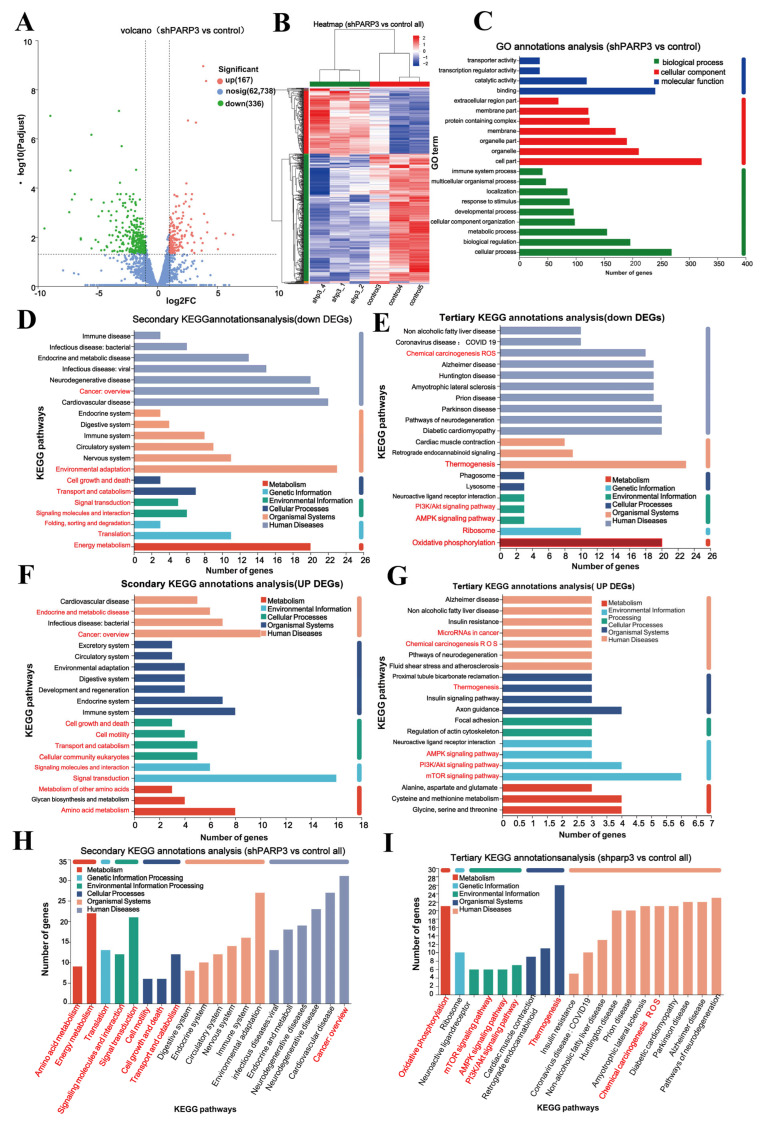
RNA sequencing analysis of the biological function of *PARP3* in AML. (**A**) Volcano plot analysis of differentially expressed genes (DEGs) between *PARP3*-knockdown and control MOLM13 cells. (**B**) Heatmap analysis of DEGs between *PARP3*-knockdown and control MOLM13 cells. (**C**) Gene ontology analysis of DEGs in *PARP3*-knockdown versus control MOLM13 cells. (**D**) Secondary KEGG pathway enrichment analysis of downregulated DEGs in *PARP3*-knockdown versus control MOLM13 cells. (**E**) Tertiary KEGG pathway enrichment analysis of downregulated DEGs in *PARP3*-knockdown versus control MOLM13 cells. (**F**) Secondary KEGG pathway enrichment analysis of upregulated DEGs in *PARP3*-knockdown versus control MOLM13 cells. (**G**) Tertiary KEGG pathway enrichment analysis of upregulated DEGs in *PARP3*-knockdown versus control MOLM13 cells. (**H**) Secondary KEGG pathway enrichment analysis of all DEGs in *PARP3*-knockdown versus control MOLM13 cells. (**I**) Tertiary KEGG pathway enrichment analysis of all DEGs in *PARP3*-knockdown versus control MOLM13 cells.

**Figure 6:**
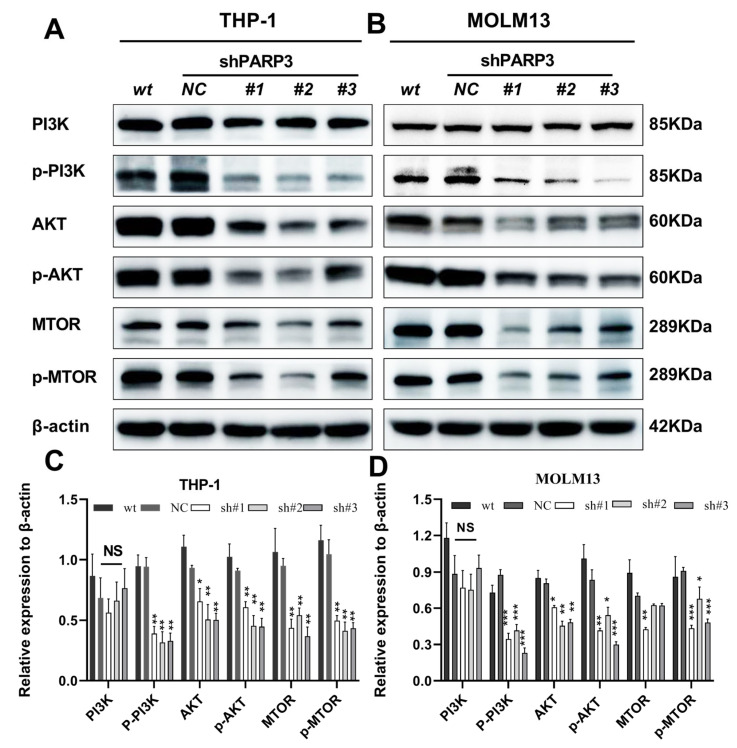
*PARP3* knockdown weakens the PI3K/AKT/mTOR signaling pathway in AML cells. Western blot assays were conducted to measure pPI3K, PI3K, pAKT, AKT, pmTOR, and mTOR protein expression in AML cells transfected with shPARP3-NC and shPARP3#1-3. (**A,C**) Expression of PI3K/AKT/mTOR signaling pathway-associated proteins between in *PARP3* knockdown’ and control’ THP-1 cells. (**B,D**) Expression of PI3K/AKT/mTOR signaling pathway-associated proteins between in *PARP3* knockdown’ and control’ MOLM13 cells.Data are presented as the mean ± SD of three independent experiments. NS, not statistically significant, * *p* < 0.05, ** *p* < 0.01, and *** *p* < 0.001 vs. the shPARP3-NC group. AML, acute myeloid leukemia; SD, standard deviation; wt, wildtype—no lentivirus transfection; NC, negative control—empty vector of lentivirus transfection; sh: short hairpin RNA.

To elucidate the mechanisms underlying the oncogenic functions of *PARP3* in AML, we conducted RNA sequencing to examine transcriptomic changes in *PARP3*-knockdown MOLM13 cells. Data processing and bioinformatic analysis were carried out using the Majorbio Cloud Platform (www.majorbio.com). Our analysis identified 27,037 expressed genes and 128,336 transcripts. Using DESeq2, we detected 167 upregulated and 336 downregulated differentially expressed genes (DEGs) following *PARP3* knockdown (Figure 5A,B). Gene Ontology (GO) enrichment analysis indicated that *PARP3* is associated with cellular organelle organization, cell motility, developmental processes, signal binding, and metabolic regulation, all of which critically contribute to tumor growth and migration (Figure 5C). Pathway analysis of the downregulated DEGs further suggested that *PARP3* knockdown predominantly suppresses the signaling pathways related to “ROS-induced carcinogenesis”, “cell growth and apoptosis”, ”signal transduction”, ”energy metabolism”, ”ROS-induced carcinogenesis”, “cellular growth and apoptosis”, “signal transduction and interaction”, and “energy metabolism” (Figure 5D). Notably, KEGG tertiary pathway enrichment analysis showed that *PARP3* downregulation was closely related to the “oxidative phosphorylation (OXPHOS)”, ”ribosome”, ”phosphatidylinositol-3-kinase/AKT (PI3K/AKT)”, and “AMPK” signaling pathways (Figure 5E). The results of the pathway enrichment analysis conducted on the upregulated DEGs indicated that *PARP3* primarily upregulated signaling pathways associated with “cancer”, “cell survival and apoptosis”, and “signal transduction and interaction”, and “energy metabolism” (Figure 5F). Moreover, KEGG tertiary pathway enrichment analysis revealed that *PARP3* upregulation was closely associated with the “mammalian target of rapamycin (mTOR)“, “PI3K/AKT”, “AMPK”, “ROS chemical carcinogenesis”, and “amino acid metabolism” signaling pathways (Figure 5G). KEGG enrichment analysis of all DEGs showed that *PARP3* was closely related to “OXPHOS”, “energy metabolism”, “cell motility”, ”adhesion”, “cell growth and death”, and ”ROS-induced carcinogenesis” (Figure 5H). Furthermore, the tertiary KEGG functional annotation analysis of DEGs showed that the most significantly enriched pathways were the “mTOR”, “PI3K/AKT”, and “AMPK” signaling pathways (Figure 5I). All of these are important factors affecting the growth and migration of tumor cells. The PI3K/AKT/mTOR signaling pathway has been widely demonstrated to promote the progression and drug resistance of AML [29,30]. To investigate whether *PARP3* plays a role in the PI3K/AKT/mTOR signaling pathway in AML, we examined the expression and phosphorylation status of key molecules in this pathway after *PARP3* knockdown. The results demonstrated that *PARP3* knockdown impaired the phosphorylation protein level of PI3K p85 in AML cells, as well as both the total and phosphorylated protein levels of the key PI3K downstream effectors AKT and mTOR, whereas there were no significant differences in the protein levels of the PI3K catalytic subunits p110δ or p110γ. (Figure 6A–D). This observation is consistent with the KEGG enrichment results from RNA sequencing. These findings suggest that *PARP3* mediates the malignant clonal expansion and progression of AML through the PI3K/AKT/mTOR pathway.

## 4. Discussion

AML remains an incurable malignant tumor that poses a significant threat to human health. Refractory and relapsed cases remain the two major causes of treatment failure [2,31]. Therefore, there is a need for new diagnostic and prognostic biomarkers to provide more options for AML treatment. In recent years, the PARP family has been reported to encompass 17 genes that encode enzymes modifying nuclear protein ADP-ribosylation, a post-translational modification critical for DNA damage response and repair [3]. Meanwhile, the PARP family has become recognized as a crucial regulator in cancer biology. [32,33]. *PARP3*, as a member of the mono-ADP-ribosyl transferase enzymes, catalyzes the post-translational modification of target proteins, mediating diverse cellular functions including the maintenance of genomic stability, epigenetic modulation, and mesenchymal transition, among others [14,34,35,36]. In recent years, *PARP3* has been reported as being overexpressed in a subset of aggressive tumors and is closely linked with poor prognosis, representing an attractive strategy to improve the sensitivity of tumors to radiotherapy and chemotherapy [19,20]. However, the role of *PARP3* in AML has not been previously investigated.

Firstly, the expression profile of *PARP3* was investigated using publicly available databases and clinical data. The results revealed that *PARP3* was overexpressed in AML and the overexpression and high expression levels correlated significantly with reduced overall survival. Furthermore, *PARP3* expression significantly correlated with advanced age and intermediate–high cytogenetic risk in AML patients and remained an independent adverse prognostic factor. Therefore, this study indicates that *PARP3* may represent a biomarker for predicting poor prognosis in AML and targeting *PARP3* may serve as a potential therapeutic target for the treatment of AML. Beyond the current study, *PARP3* has been shown to be aberrantly expressed in multiple human cancers. For example, elevated *PARP3* levels have been observed in primary glioblastoma biopsies [20]. Moreover, later studies have shown that *PARP3* expression is positively correlated with the invasive basal-like and mesenchymal subtypes of breast cancer, where is is significantly increased [19,36].

Next, to explore the biological functions of *PARP3* in the AML, *PARP3*-knockdown AML cells were established using lentiviral transduction. The results indicate that specifically downregulating *PARP3* expression impaired AML cell proliferation, disrupted cell cycle progression, inhibited migration, and induced apoptosis. Specifically, this disruption is manifested as a decrease in the fraction of cells in the S phase of the cells and an increase in the fraction of cells in the G0/G1 phase. Therefore, *PARP3* may be involved in the mitotic process of AML, and inhibiting *PARP3* could lead to mitotic defects, thereby inducing apoptosis. Research has consistently demonstrated that inhibition of *PARP3* potentiates the efficacy of the vinca alkaloid vinorelbine against metastatic breast cancer through increased mitotic abnormalities and apoptosis [21]. Previous studies have indicated that the protein targets of *PARP3* include mitotic components such as NuMa and Tankyrase 1, DNA repair proteins such as Ku80 and PARP1, as well as histone H2B, particularly Glu2 [14,15,37,38]. These findings support the notion that targeting *PARP3* can disrupt cell mitosis and induce apoptosis, which is consistent with our experimental results.

Subsequently, our study confirmed that the migration ability of AML cells was reduced after the knockdown of *PARP3*. This finding was further verified by western blot assays. *PARP3* knockdown significantly suppresses the metastasis-promoting proteins Vimentin and N-cadherin, while concomitantly upregulating the expression of the epithelial tumor suppressor E-cadherin. Being a liquid tumor, AML exhibits inherently higher constitutive motility and invasive potential than solid tumors. Nevertheless, AML cells still require robust molecular mechanisms to invade other bone marrow sites or establish extramedullary implantations [39]. Recent studies have demonstrated epithelial-to-mesenchymal transition (EMT)-related genes such as N-cadherin [40], TWIST1 [26], and Snail1 [27] are strongly associated with prognosis, migration, and extramedullary infiltration in AML. N-cadherin and Vimentin play critical roles in cell adhesion and motility, thereby enhancing the potential for AML cells to invade extramedullary tissue [41]. Therefore, targeting *PARP3* to modulate AML-associated EMT represents a promising therapeutic strategy warranting further investigation.

In this study, RNA-seq analysis revealed that *PARP3* was correlated with oxidative phosphorylation, migratory pathways, cellular growth/death, the AMPK signaling pathway, the PI3K/AKT/mTOR signaling pathway, and ROS. Dysregulation of the PI3K/AKT/mTOR pathway is a fundamental driver of oncogenesis and tumor advancement [42,43]. Indeed, the PI3K/AKT/mTOR pathway is dysregulated in approximately 50% of tumors [44], making it the most frequently activated pathway in human cancers. Therefore, the PI3K/AKT/mTOR pathway was selected for experimental validation in our cellular models. To examine the effect of *PARP3* knockdown on signaling, we performed cell experiments in MOLM13 and THP-1 cells. The results showed that *PARP3* depletion inhibited the PI3K/AKT/mTOR pathway. In particular, the level of phosphorylated protein was significantly reduced, whereas there were no significant differences in the protein levels of the PI3K catalytic subunits p110δ or p110γ. Regarding why PI3K’s downstream substrates were suppressed without changes in the protein expression level of the PI3K p110 subunit after *PARP3* knockdown, we speculate that epigenetic alterations or changes in the post-translational modifications of PI3K may have occurred following *PARP3* knockdown. Interestingly, as an enzyme known to catalyze ADP-ribosylation, *PARP3* primarily functions by modifying target proteins through ADP-ribosylation. It remains unclear whether the PI3K p110 subunit itself is directly ADP-ribosylated by *PARP3*, or whether other yet unidentified proteins modified by *PARP3* may indirectly regulate the PI3K subunit. This represents one of the key directions for our future research. Moreover, it should be noted that this study only focused on validating changes in AKT, the downstream molecule of mTOR; future studies are warranted to explore whether *PARP3* also influences other mTOR downstream effectors, such as p70S6K, eIF4E, and PKCα, which may contribute to additional functional outcomes in AML pathogenesis. Some studies have also reported the close relationship between *PARP3* and mTOR. Baker et al. indicated that *PARP3* is involved in TGFβ-induced EMT through the Rictor/mTORC2 signaling pathway in BRCA1-deficient triple-negative breast cancer (TNBC) cells [45]. José-Manuel Rodriguez-Vargas et al. demonstrated that *PARP3* drives astrocytic differentiation by modulating Nox4-mediated ROS generation, which in turn is critical for the activation of mTORC2 [46]. The PI3K/AKT/mTOR pathway is often hyperactivated in the AML to support the invasive proliferation of AML [30,47]. Increasing evidence reveals that targeting the PI3K/AKT/mTOR pathway may serve as an effective strategy to treat AML [48,49]. However, inhibitors of the PI3K/AKT/mTOR pathway have not yet been translated into clinical practice, likely due to the compensatory activation of other survival pathways. Therefore, this study can provide new insights for inhibiting the PI3K/AKT/mTOR signaling pathway.

## 5. Conclusions

*PARP3* drives AML cell proliferation and migration through activation of the PI3K/AKT/mTOR pathway, highlighting its potential role in AML pathogenesis. Targeting *PARP3* may achieve a potential anti-leukemia effect by disrupting migration and cell growth. However, there are other pathways such as oxidative phosphorylation, ROS, and energy metabolism that still need to be extensively explored, which may guide our subsequent investigations.

## Figures and Tables

**Table 1:** Primer sequences.

Genes	Sequences (5′-3′)
*PARP3*	Forward: GACCAACATCGAGAACAACAACA
	Reverse: GCCTTGTGAAGTGGTTGATCT
*β-actin*	Forward: CACCCAGCACAATGAAGATCAAG
	Reverse: TCATAGTCCGCCTAGAAGCATTT

Note: *PARP3*,Poly(ADP-ribose)polymerase 3.

**Table 2:** Characterization of clinical features in TCGA AML patients based on *PARP3* low/high expression groups.

Clinical Parameters	*PARP3* Low	*PARP3* High	*p*
Sex, male/female	38/37	46/30	0.372
Age, years (range)	49(21–81)	58(21–88)	0.001 *
WBC, ×109/L (range)	34(1–224)	38(1–172)	0.748
BM, % (range)	41(0–97)	38(0–97)	0.205
PB, % (range)	64(0–98)	69(0–100)	0.378
Gene mutations ^#^			
*NPM1*, wildtype/mutant	66/9(12%)	67/9(11.8%)	0.493
*FLT3*, wildtype/mutant	57/18(24%)	53/23(30%)	0.468
*IDH1/IDH2*, wildtype/mutant	73/2(3%)	73/3(4%)	0.437
*NRAS/KRAS*, wildtype/mutant	70/5(7%)	74/2(3%)	0.194
Cytogenetic classification ^#^			0.017 *
Favorable	25(16.6%)	5(3.3%)	
Intermediate	31(20.5%)	52(34.4%)	
Poor	19(12.6%)	19(12.6%)	
Favorable vs. Intermediate/poor			0.001 *
ELN risk stratification ^#^			0.011 *
Favorable	21(13.9%)	5(3.3%)	
Intermediate	41(27.2%)	55(36.4%)	
Poor	13(8.6%)	16(10.6%)	
Favorable vs. Intermediate/poor			0.001 *
FAB ^#^			0.576
M0	5(3.3%)	10(6.6%)	
M1	17(11.2%)	18(11.9%)	
M2	22(14.6%)	16(10.6%)	
M3	14(9.3%)	0(0)	
M4	13(8.6%)	16(10.6%)	
M5	3(2.0%)	12(8.0%)	
M6	0(0)	2(1.3%)	
M7	1(0.7%)	2(1.3%)	
OS, years	2.26 (0–7.84)	0.87 (0–4.58)	<0.0001 *

AML = acute myeloid leukemia; WBC = white blood cells; BM = bone marrow blast; PB = peripheral blood leukemia cell count; ELN = The European Leukemia Net; FAB = French–American–British classification; OS = overall survival. ^#^ Percentage was defined as (number of events/total cases) per group. * *p* < 0.05 indicates statistical significance.

**Table 3:** Univariate and Multivariate of Cox regression analysis for overall survival in AML patients.

	OS			OS	
	Univariate Analysis			Multivariate Analysis	
	Hazard Ratio (95% CI)	*p* Value		Hazard Ratio (95% CI)	*p* Value
WBC	1.005 (1.000–1.009)	0.034 *	WBC	1.008 (1.003–1.013)	0.001 *
Age	1.038 (1.023–1.054)	0.000 *	Age	1.035 (1.019–1.052)	0.000 *
Sex	0.996 (0.654–1.517)	0.986	*PARP3*	1.035 (1.011–1.059)	0.004 *
*PARP3*	3.190 (2.044–4.979)	0.000 *	ELN risk		0.015 *
*NPM1*	1.011 (0.537–1.903)	0.972	Favorable vs. intermediate	2.924 (0.455–18.771)	0.258
*FLT3*	1.226 (0.766–1.962)	0.395			
*IDH1/IDH2*	0.301 (0.042–2.161)	0.232	Intermediate vs. adverse	0.394 (0.177–0.880)	0.023 *
*NRAS/KRAS*	0.543 (0.171–1.723)	0.300			
ELN risk	1.781 (1.266–2.507)	0.001 *	Favorable vs. adverse	0.485 (0.285–0.826)	0.008 *

COX regression analysis was used. AML = acute myeloid leukemia, OS = overall survival, CI = confidence interval, WBC = white blood cells. ELN = The European Leukemia Net. In all AML patients, through Univariate/Multivariate Cox analysis, Age, WBC, *PARP3*, ELN risk were independent markers of poorer OS in the AML (*p* < 0.05, respectively), * *p* < 0.05, with statistical significance.

## Data Availability

The RNA expression data, clinical and laboratory parameter data, gene mutation data, and the survival data of 151 newly diagnosed AML patients in the TCGA-AML cohorts were downloaded from the Cancer Genome Atlas (TCGA) database (https://portal.gdc.cancer.gov/, accessed on 17 January 2024). GSE13159, GSE15061, and GSE14468 was obtained from the GEO database (https://www.ncbi.nlm.nib.gov/geo/, accessed on 17 January 2024). The original contributions presented in this study are included in the article/Supplementary Materials. Further inquiries can be directed to the first author.

## Supplementary Materials

The following supporting information can be downloaded at: https://www.mdpi.com/article/10.3390/cancers17183076/s1, Figure S1: *PARP3* promotes AML cell proliferation in vitro; Figure S2: Original western blot images; Table S1: Characteristics of primary AML samples and healthy controls used for these experiments.

## Abbreviations

*PARP3*	Poly(ADP-ribose)polymerase 3
AML	Acute myeloid leukemia
FLT3-ITD	FMS-related receptor tyrosine kinase 3 internal tandem duplications
EMT	Epithelial-to-mesenchymal transition
