# Supplementary material for: PARP3 Promotes AML Progression via Activation of PI3K/AKT/mTOR Signaling
Source: Cancers (Basel). 2025 Sep 20;17(18):3076. doi: 10.3390/cancers17183076 (PMC12468979; doi:10.3390/cancers17183076)
Supplement: Supplementary file 1 [file cancers-17-03076-s001.zip › Supplementary Figure S1 & Table S1.pdf]

Supplementary Table S1. Characteristics of primary AML samples and healthy controls used for these studies.

Number	Gender	Age	FAB Subtype	Karyotype	Molecular Features
AML1	male	24	M2	46, XY, t (8; 21) (q22; q22) [9]	RUNX1-RUNX1；ASXL1
AML2	male	59	M4	47，XY[10]	U2AF1
AML3	male	68	M2	45, XY, −7 [20]	-
AML4	female	53	M2b	46,XX,t(8:21)(q22;q22)[5]	KIT; RUNX1-RUNX1
AML5	male	44	M5	46, XY [20]	-
AML6	male	66	M5	N/A	N/A
AML7	male	34	M2	46, XY [20]	NRAS, KRAS
AML8	female	71	M2	46, XX [20]	WT1
AML9	female	44	M4	46, XX [20]	NRAS
AML10	male	17	M1	46, XY, t(8;21)(q22;q22)[17]	RUNX1-RUNX1,KIT
AML11	male	39	MDS-M6	46, XY [20]	NRAS
AML12	female	40	M2	46, XX [20]	-
AML13	female	61	MDS-M2	46, XX, t(8;21)(q22;q22)[15]	FLT3-ITD , RUNX1-RUNX1
AML14	female	80	M4	46, XX [20]	-
AML15	female	20	M2	46, XX [20]	TET2, WT1
AML16	male	56	M2	46, XY [20]	-
AML17	female	53	M5	46, XX, t(9;11)(p23;q23)[20]	KMT2A-MLLT3;FLT3-ITD
AML18	female	57	M4	46, XX,del(2)(q31),inv(16)(p13q22)[20]	CEBPA;CBFB/MYH11
AML19	male	37	M1	46, XY [20]	-
AML20	male	43	M2	46, XY [20]	-
AML21	female	68	M5	N/A	IDH2;TP53;SF3B1
AML22	female	67	N/A	46, XX [20]	-
AML23	male	65	M3	46, XY, t(15;17)(q24;q21)[20]	PML-RARA
Control-1	Male	23			
Control-2	female	24			
Control-3	Male	56			
Control-4	female	47			
Control-5	female	65			
Control-6	Male	26			
Control-7	Male	38			
Control-8	female	49			
Control-9	female	53			
Control-10	Male	32			
Control-11	female	28			

supplementary FigureS1

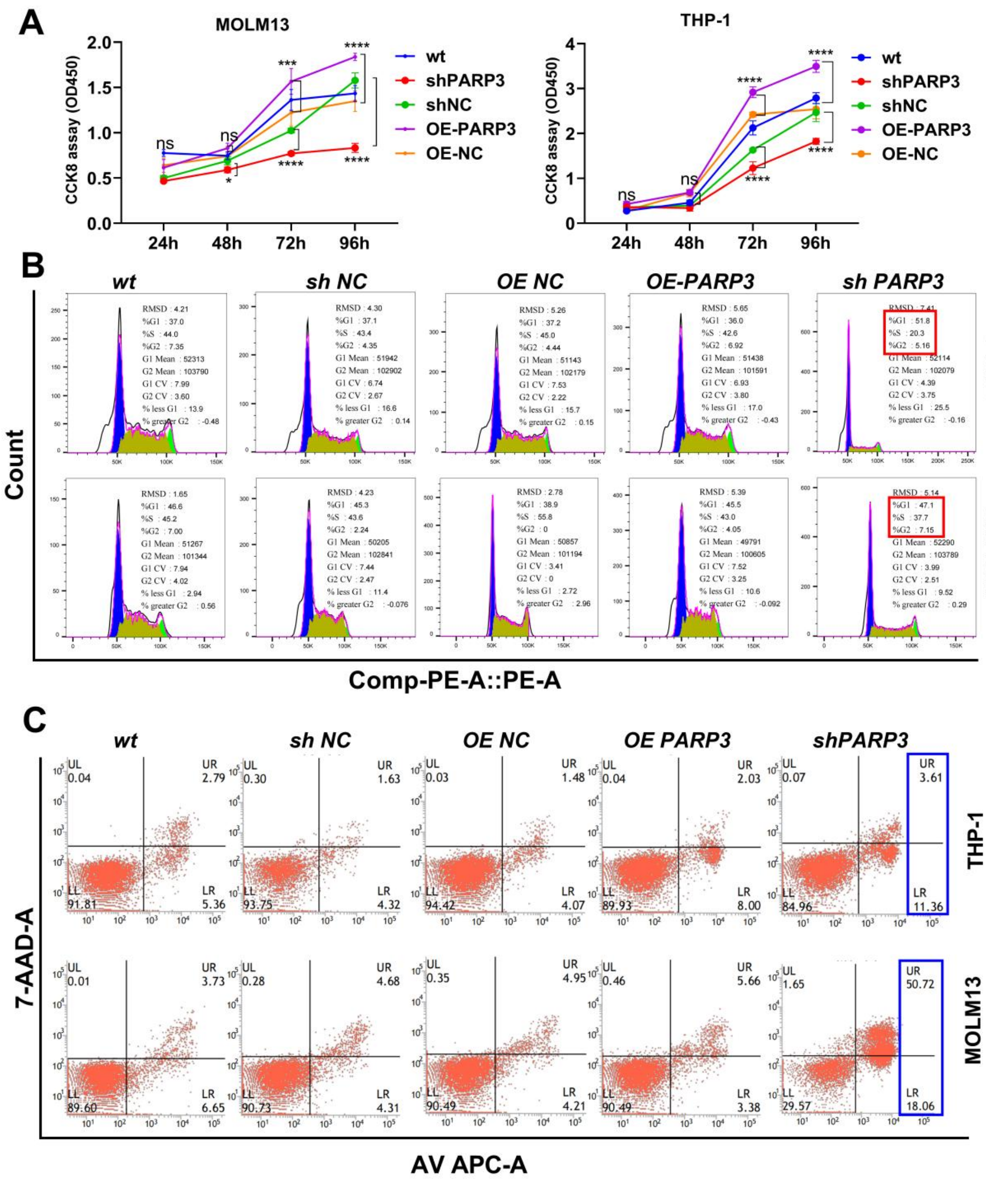

Figure S1. PARP3 promotes AML cell proliferation in vitro. **(A)** PARP3 over-expression promoted MOLM13 and THP-1 cell proliferation which were demonstrated by using CCK-8 . **(B-C)** There no significant differences in apoptosis and Cell cycle were observed between the PARP3 upregulated and control MOLM13 and THP-1 cells. Data are presented as mean ± SD of three independent experiments. *P<0.05, **P<0.01, ***P<0.001 and ****P＜0.0001 vs. the sh-PARP3-NC group. AML, Acute myeloid leukemia; CCK, cell counting kit; SD, standard deviation.wt,Wild Type,no lentiviruses transfection ; NC, negative control,empty vector of lentiviruses transfection; sh:short hairpin RNA;OE: over expression.
