# Supplementary material for: PARP3 Promotes AML Progression via Activation of PI3K/AKT/mTOR Signaling
Source: Cancers (Basel). 2025 Sep 20;17(18):3076. doi: 10.3390/cancers17183076 (PMC12468979; doi:10.3390/cancers17183076)

Original Images

1. Efficacy of PARP3 knockdown in THP-1 and MOLM13 cells by lentiviruses transfection was detected using WB (Original images)

THP-1

Figure2A THP1

wt sh-NC sh1 sh2 sh3

PARP3 60KDa

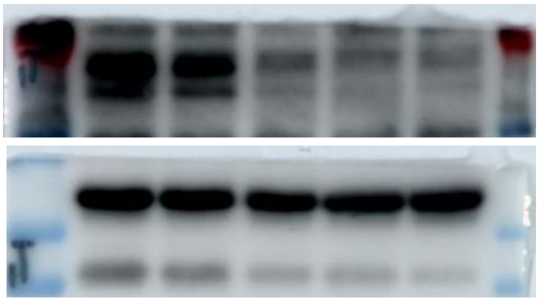

β-actin 42KDa

Second repetition

wt sh-NC sh1 sh2 sh3

PARP3 60KDa

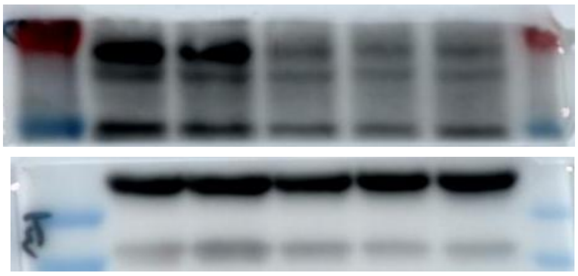

β-actin 42KDa

Third repetition

wt sh-NC sh1 sh2 sh3

PARP3 60KDa

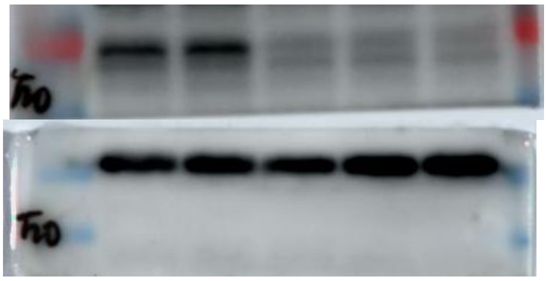

β-actin 42KDa

Figure 2A MOLM13

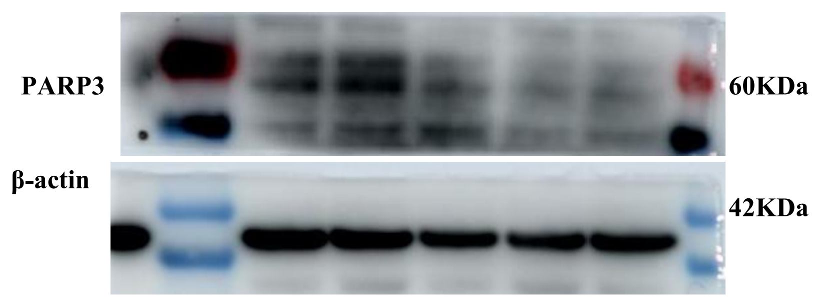

Second repetition

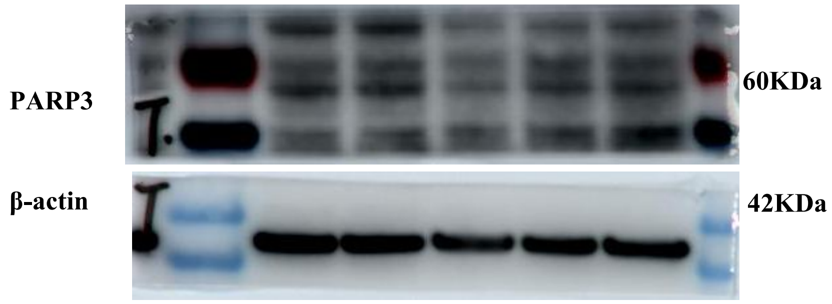

Third repetition

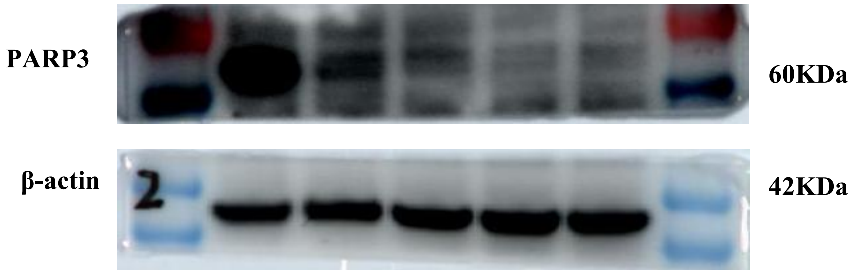

2. **Expression of cell apoptosis-associated protein BCL2 and BAX in PARP3 knockdown and control group in AML cells.**

THP-1

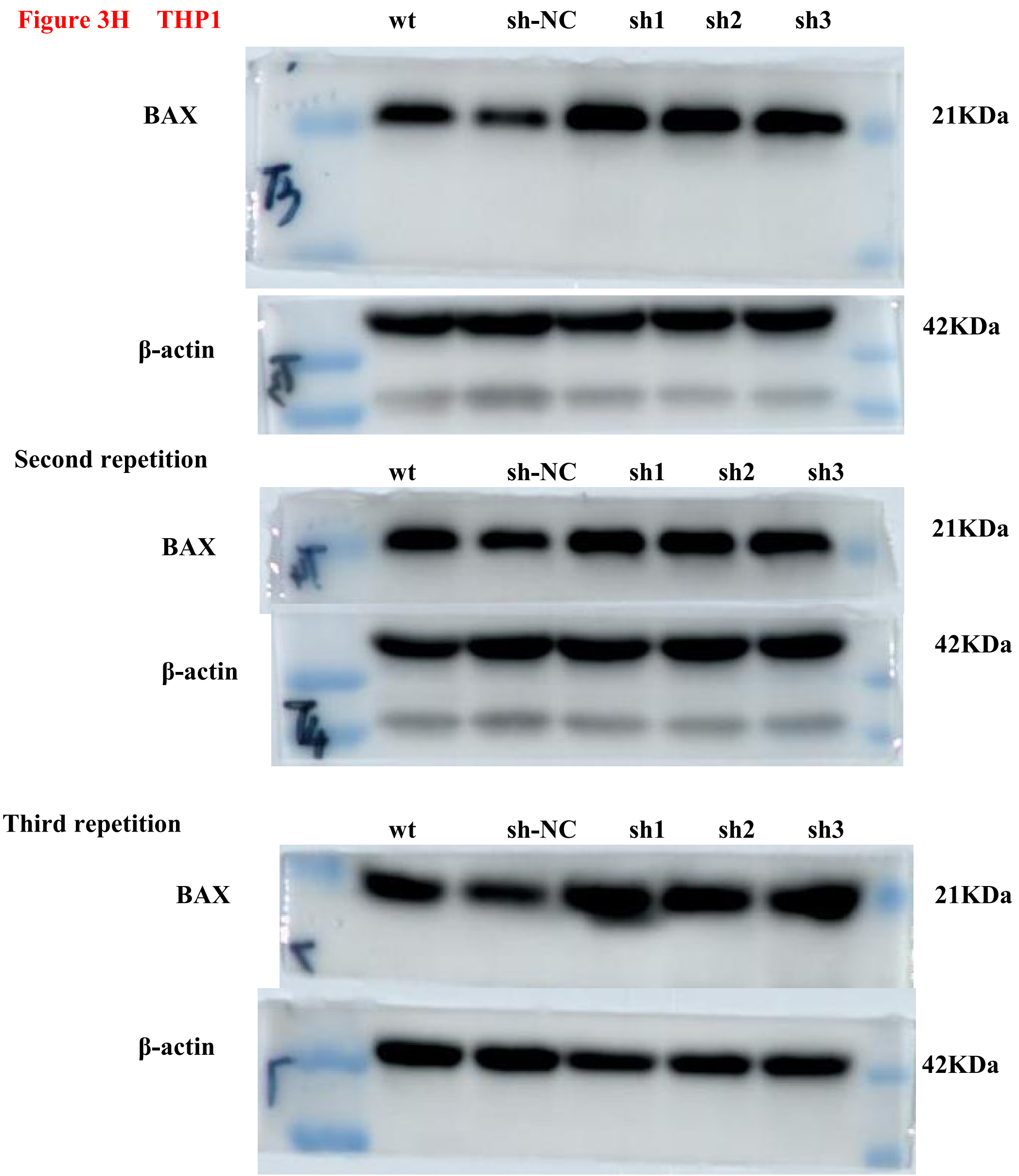

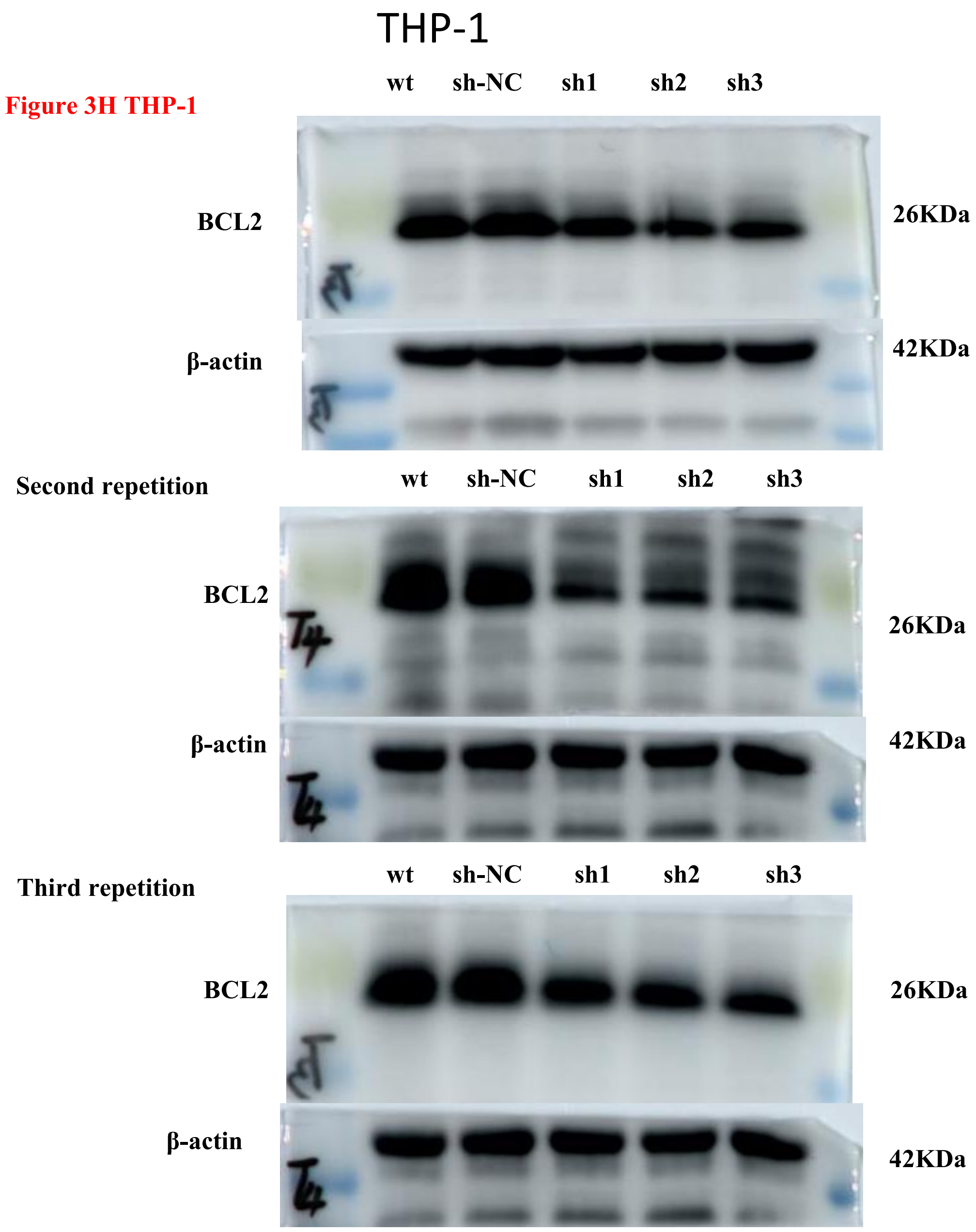
THP-1
Figure 3H THP-1
wt sh-NC sh1 sh2 sh3
BCL2
26KDa
β-actin
42KDa
Second repetition
wt sh-NC sh1 sh2 sh3
BCL2
26KDa
β-actin
42KDa
Third repetition
wt sh-NC sh1 sh2 sh3
BCL2
26KDa
β-actin
42KDa

Figure 3F MOLM13

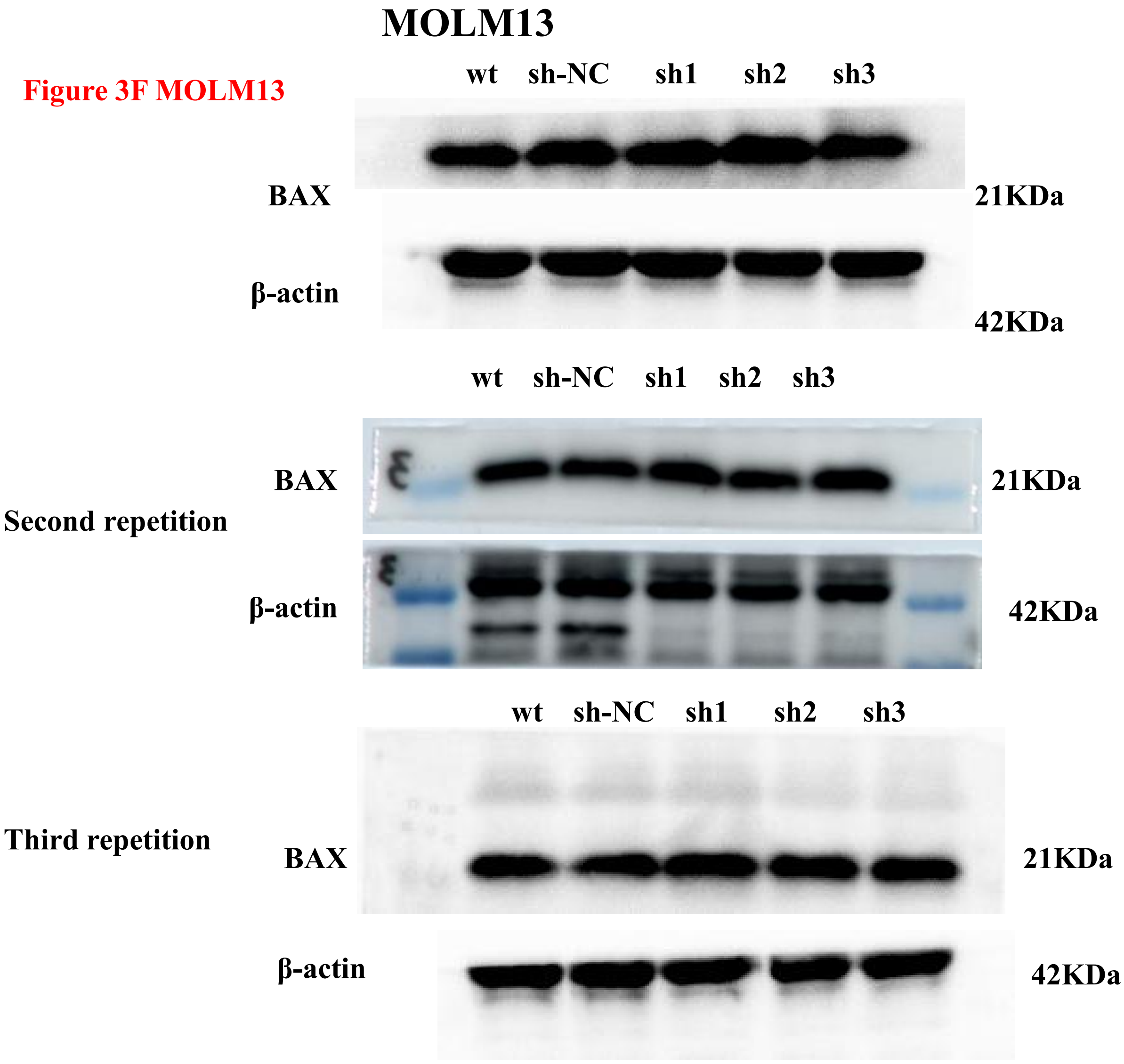

MOLM13

Figure 3F
MOLM13

Second repetition

Third repetition

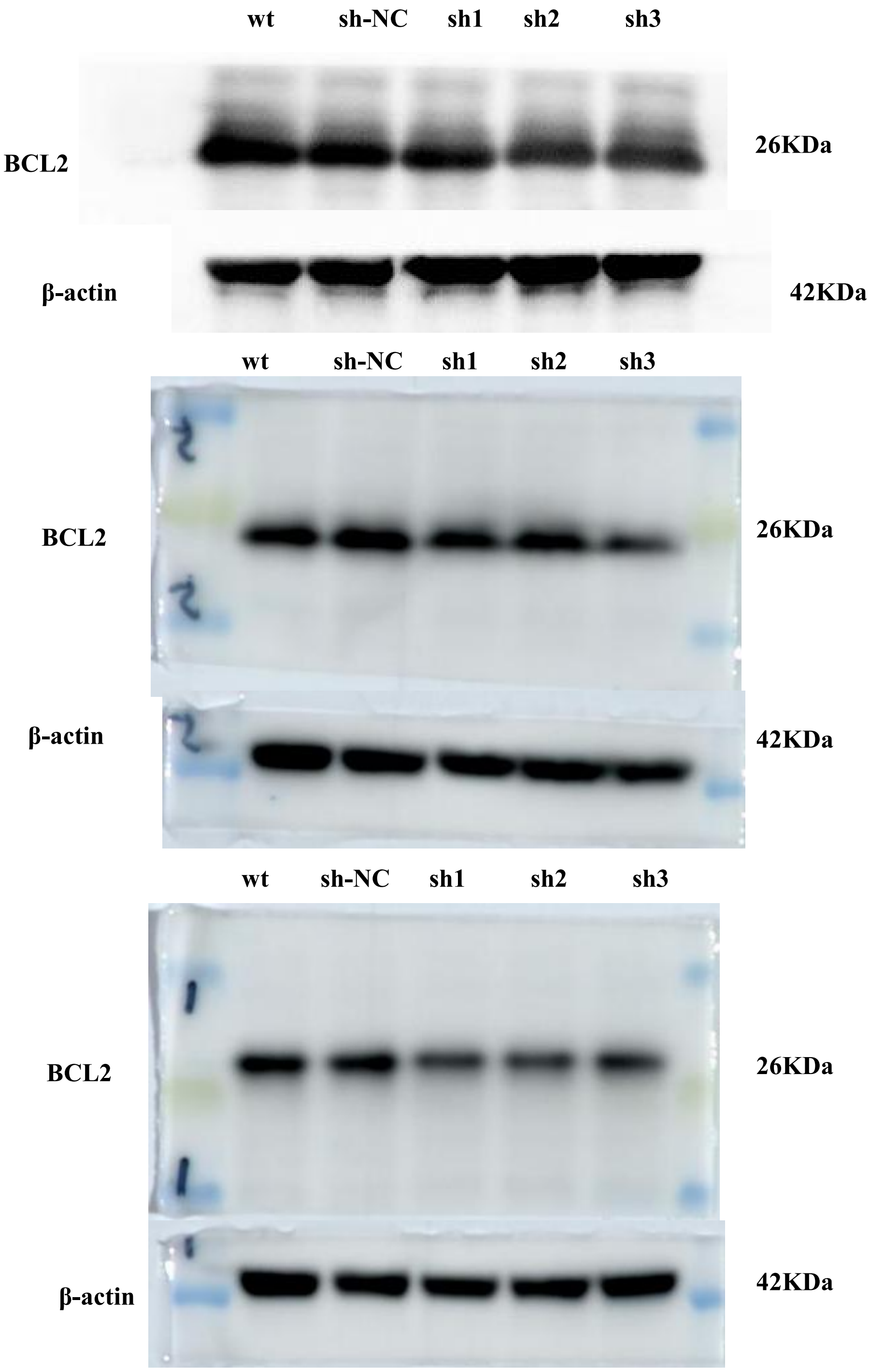

3 Expression of cell EMT-associated protein E-cadherin, N-cadherin, Vimentin, Snail ,and TWIST1 in PARP3 knockdown and control group in AML cells. (Original images)

Figure 4B THP1

wt sh-NC sh1 sh2 sh3

N-cadherin 150KDa

β-actin 42KDa

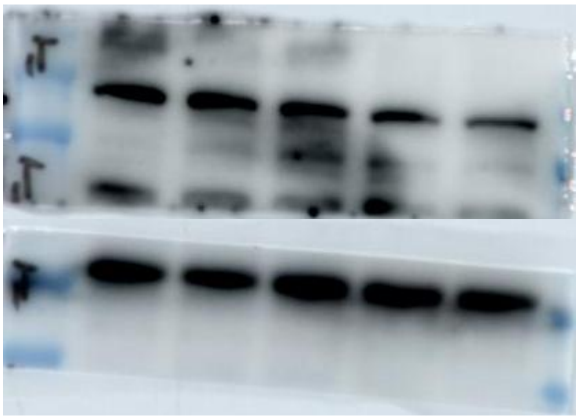

Second repetition

wt sh-NC sh1 sh2 sh3

N-cadherin 150KDa

β-actin 42KDa

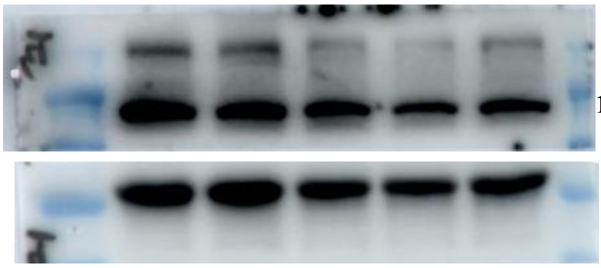

Third repetition

wt sh-NC sh1 sh2 sh3

N-cadherin 150KDa

β-actin 42KDa

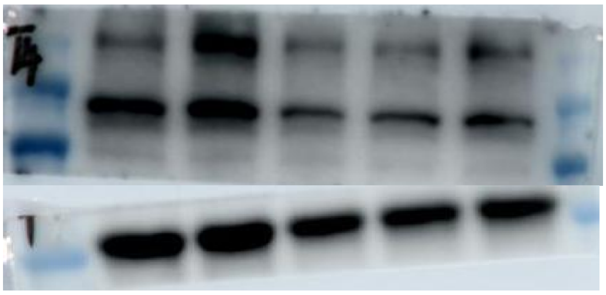

THP-1

Figure 4B THP1

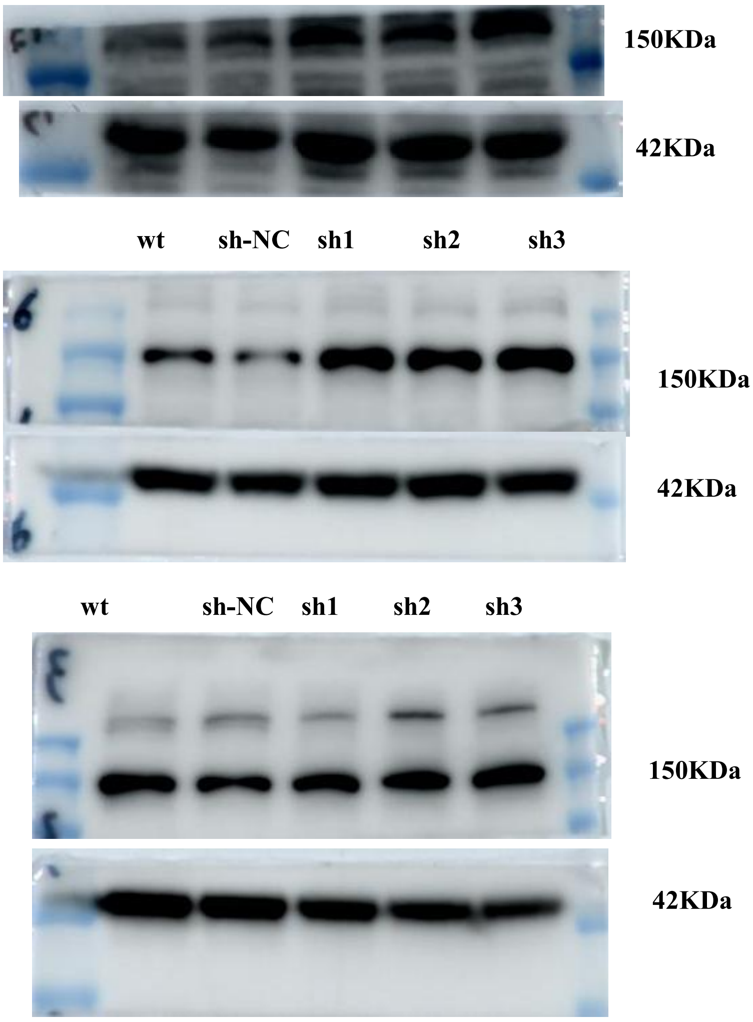

THP-1

Figure 4B THP1

wt sh-NC sh1 sh2 sh3

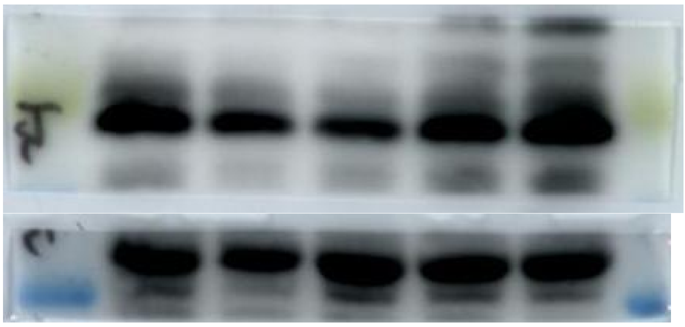

TWIST1 **26KDa**

β-actin **42KDa**

Second repetition

wt sh-NC sh1 sh2 sh3

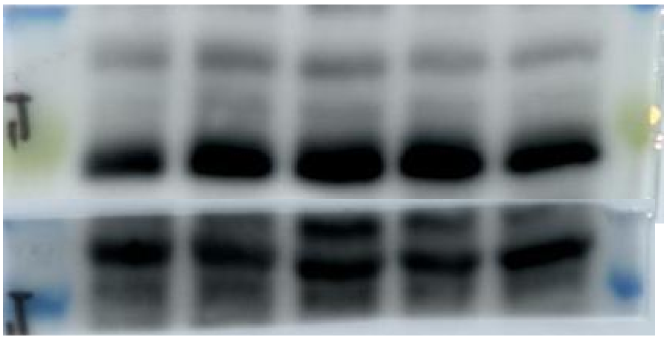

TWIST1 **26KDa**

β-actin **42KDa**

Third repetition

wt sh-NC sh1 sh2 sh3

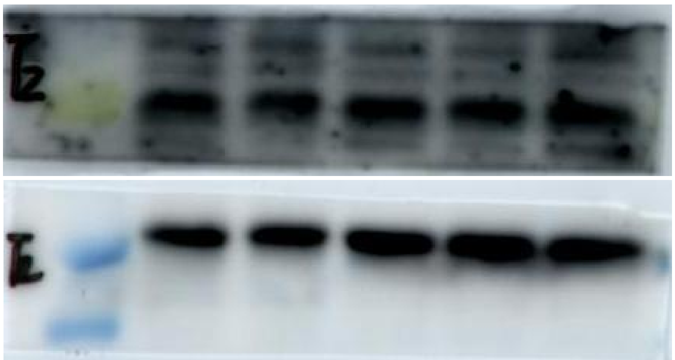

TWIST1 **26KDa**

β-actin **42KDa**

Figure 4B THP1

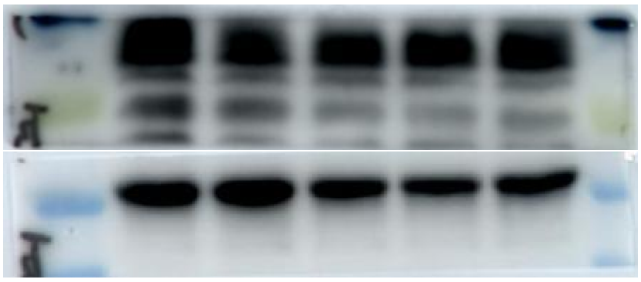

Second repetition

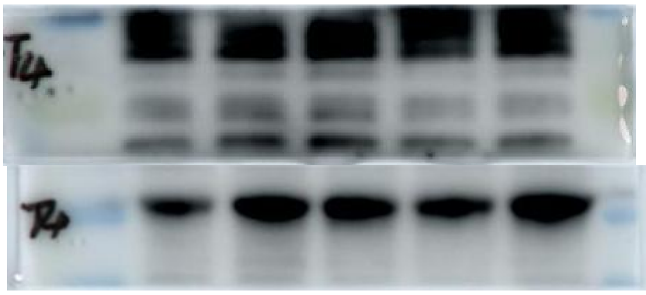

Third repetition

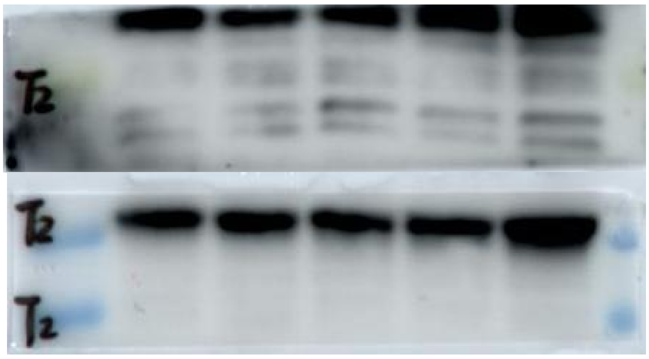

THP-1

Figure 4B THP-1

wt sh-NC sh1 sh2 sh3

Vimentin

β-actin

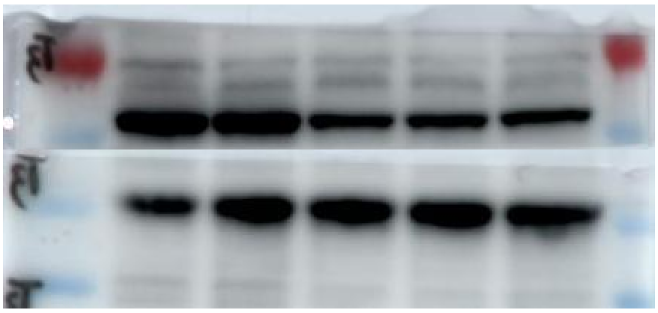

54KDa

42KDa

Second repetition

wt sh-NC sh1 sh2 sh3

Vimentin

β-actin

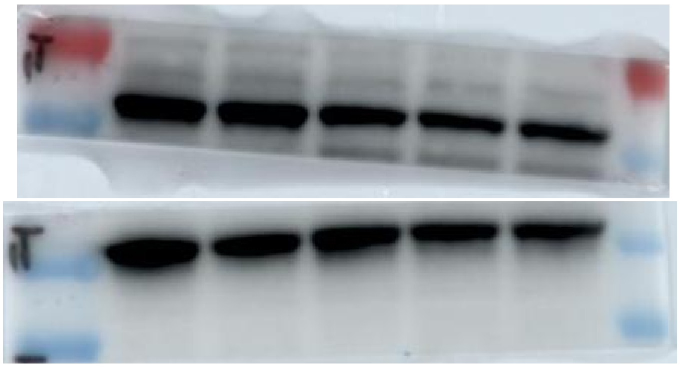

54KDa

42KDa

Third repetition

wt sh-NC sh1 sh2 sh3

Vimentin

β-actin

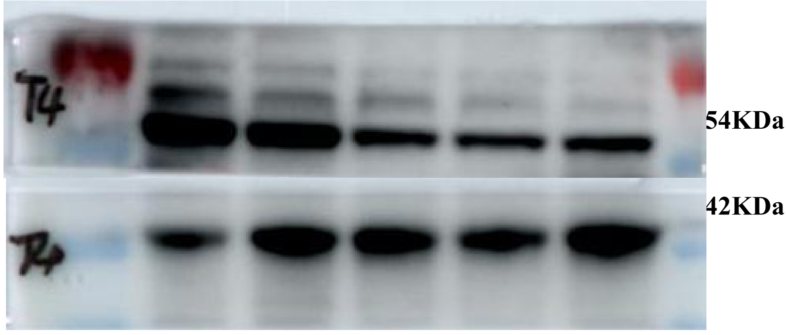

54KDa

42KDa

Figure 4B MOLM13

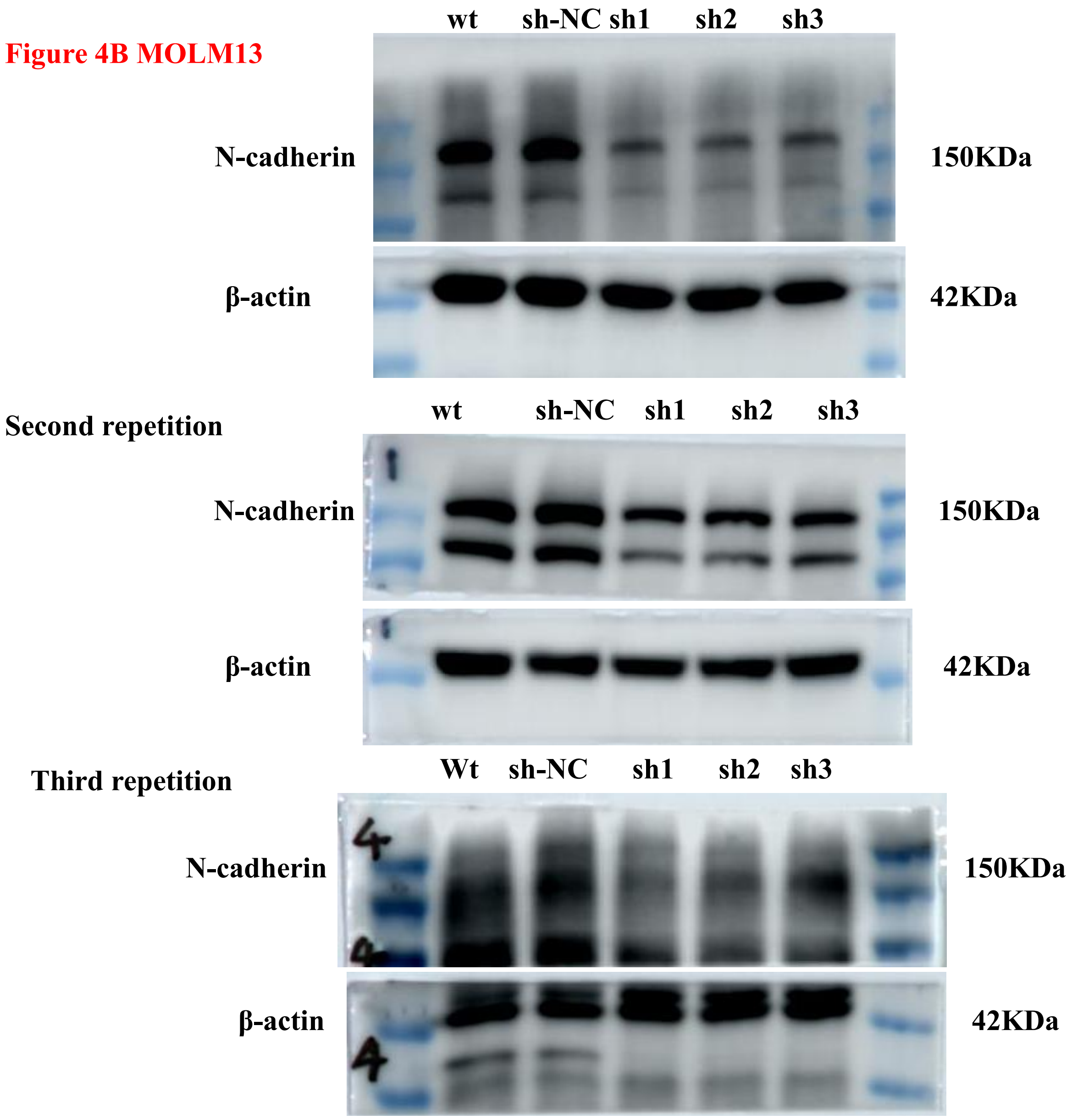

Figure 4B MOLM13

wt sh-NC sh1 sh2 sh3

E-cadherin 150KDa

β-actin 42KDa

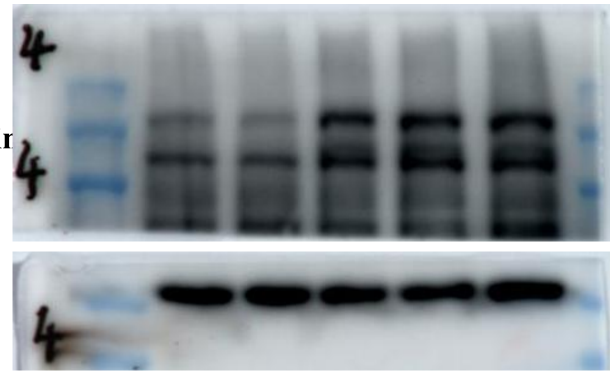

Second repetition

wt sh-NC sh1 sh2 sh3

E-cadherin 150KDa

β-actin 42KDa

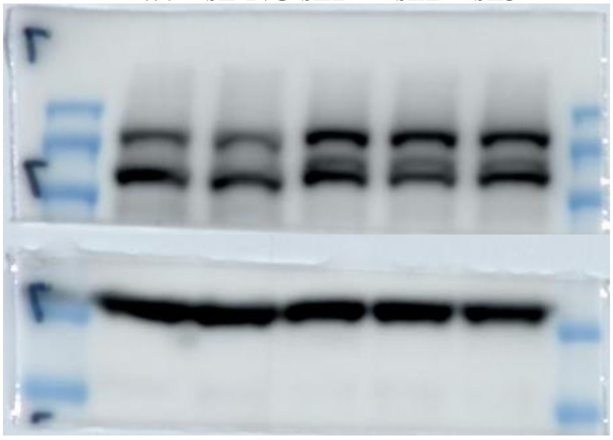

Third repetition

wt sh-NC sh1 sh2 sh3

E-cadherin 150KDa

β-actin 42KDa

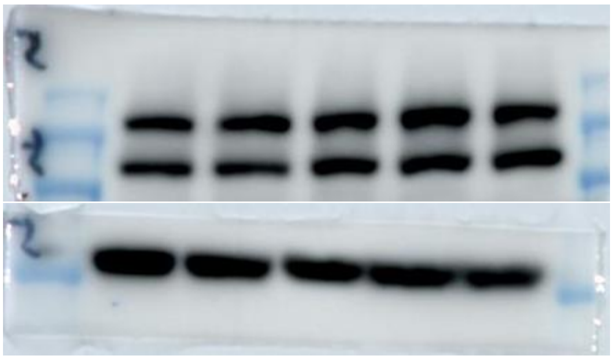

Figure 4B MOLM13

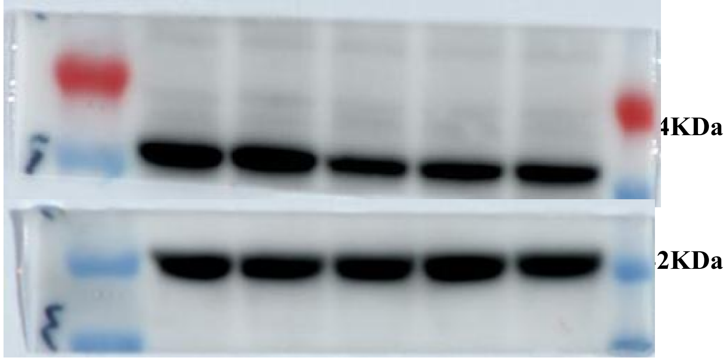

Second repetition

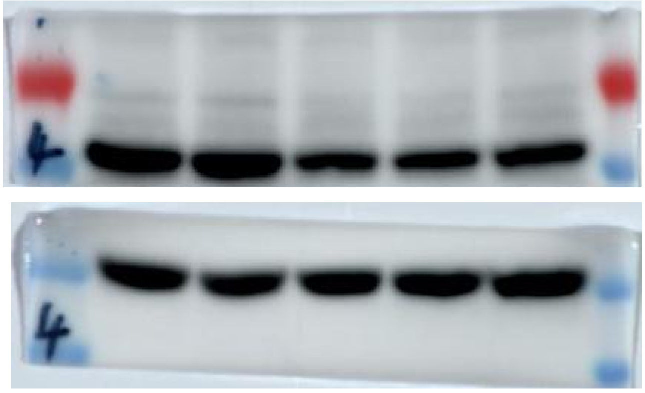

Third repetition

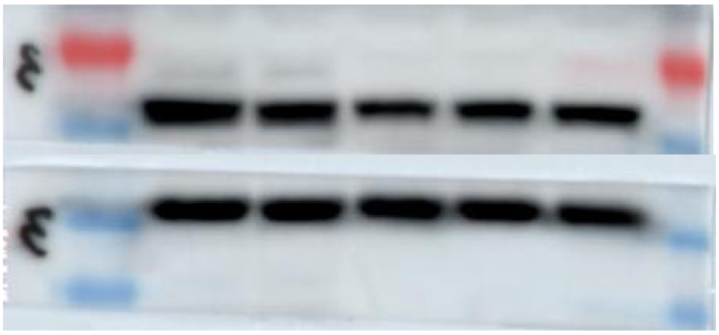

Figure 4B MOLM13

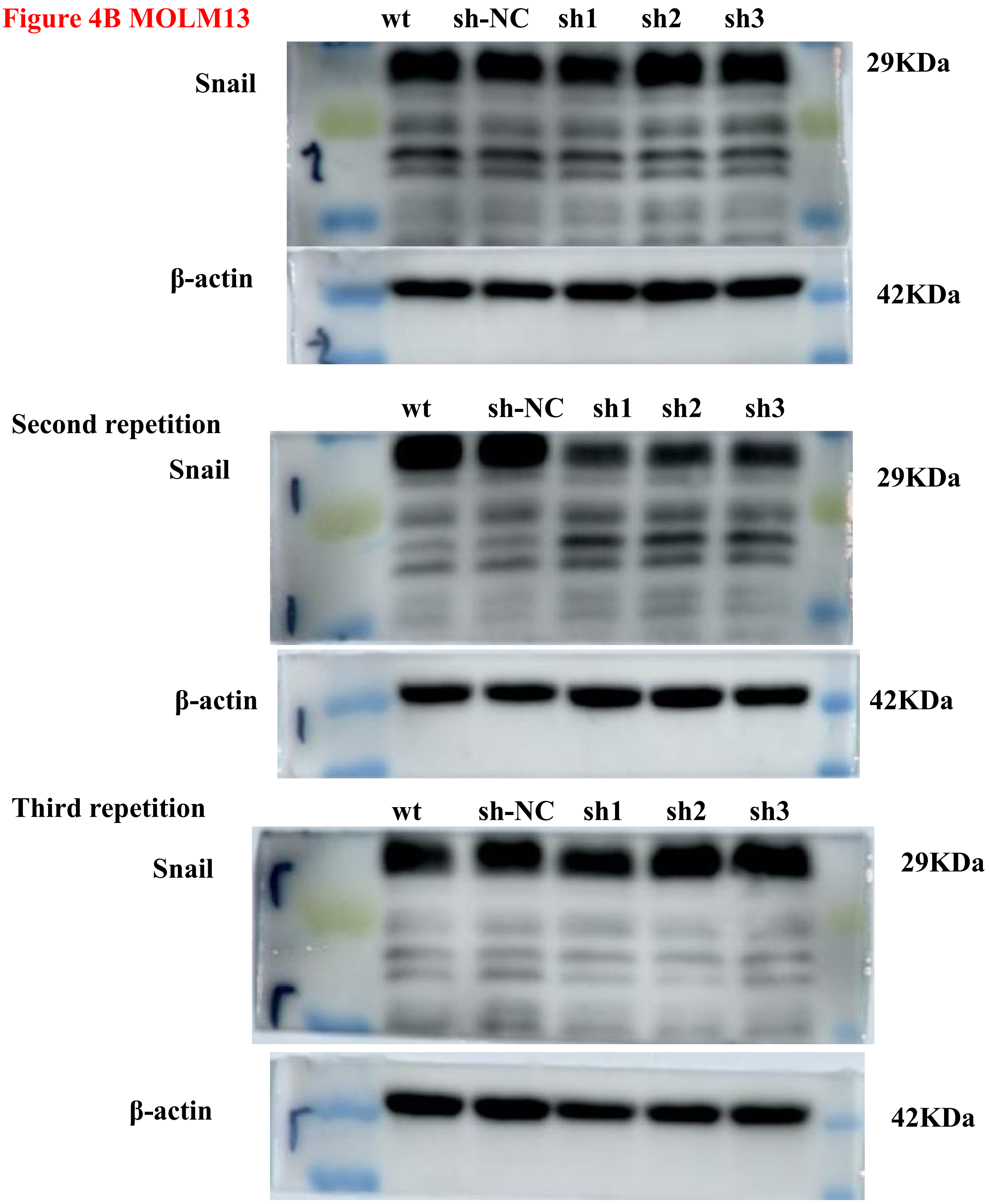

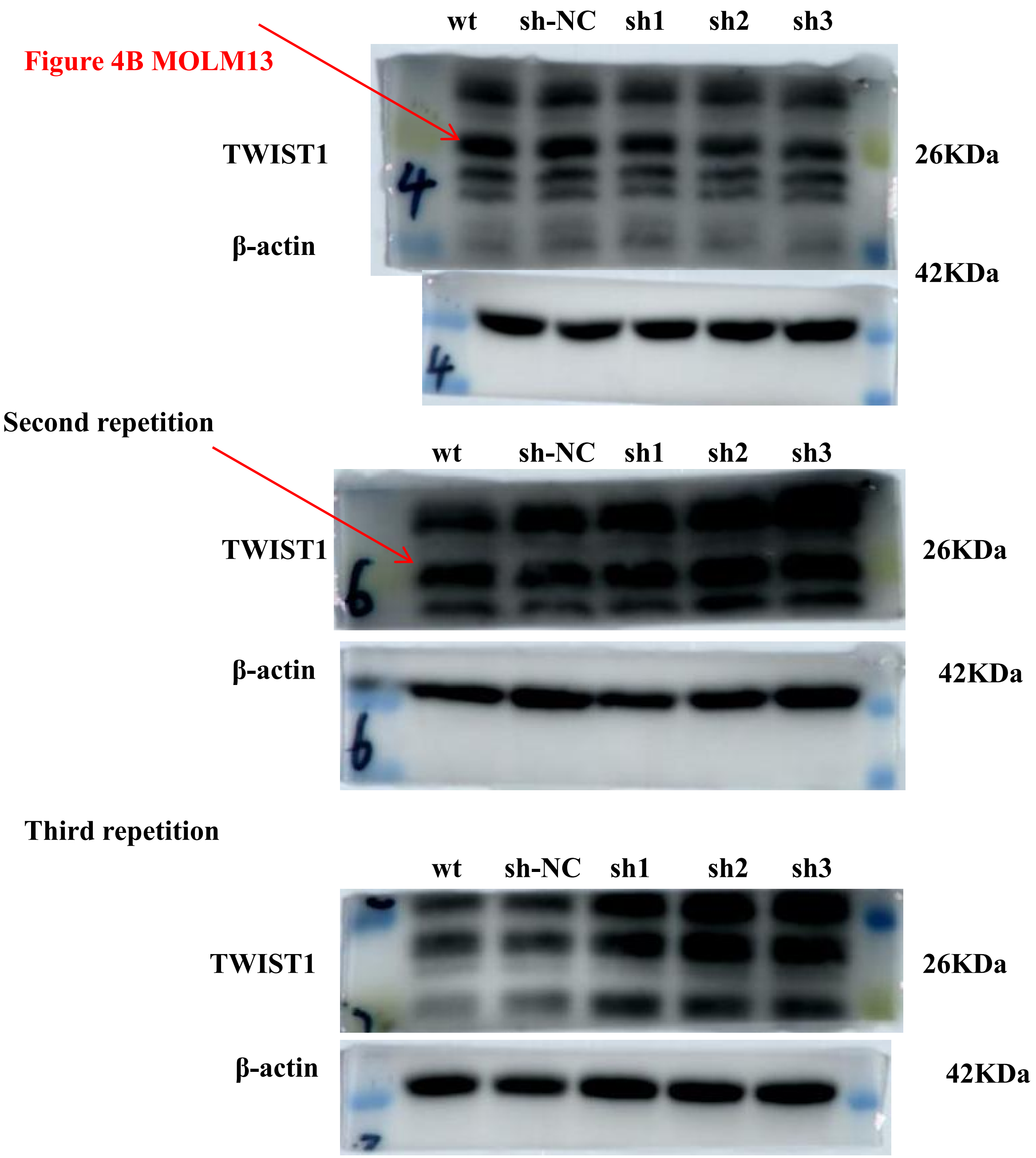
Figure 4B MOLM13
wt sh-NC sh1 sh2 sh3
TWIST1
26KDa
β-actin
42KDa
Second repetition
wt sh-NC sh1 sh2 sh3
TWIST1
26KDa
β-actin
42KDa
Third repetition
wt sh-NC sh1 sh2 sh3
TWIST1
26KDa
β-actin
42KDa

4 PARP3 knockdown suppresses the PI3K/AKT/mTOR signaling pathway of AML cells (Original Images)

Figure 6A THP1

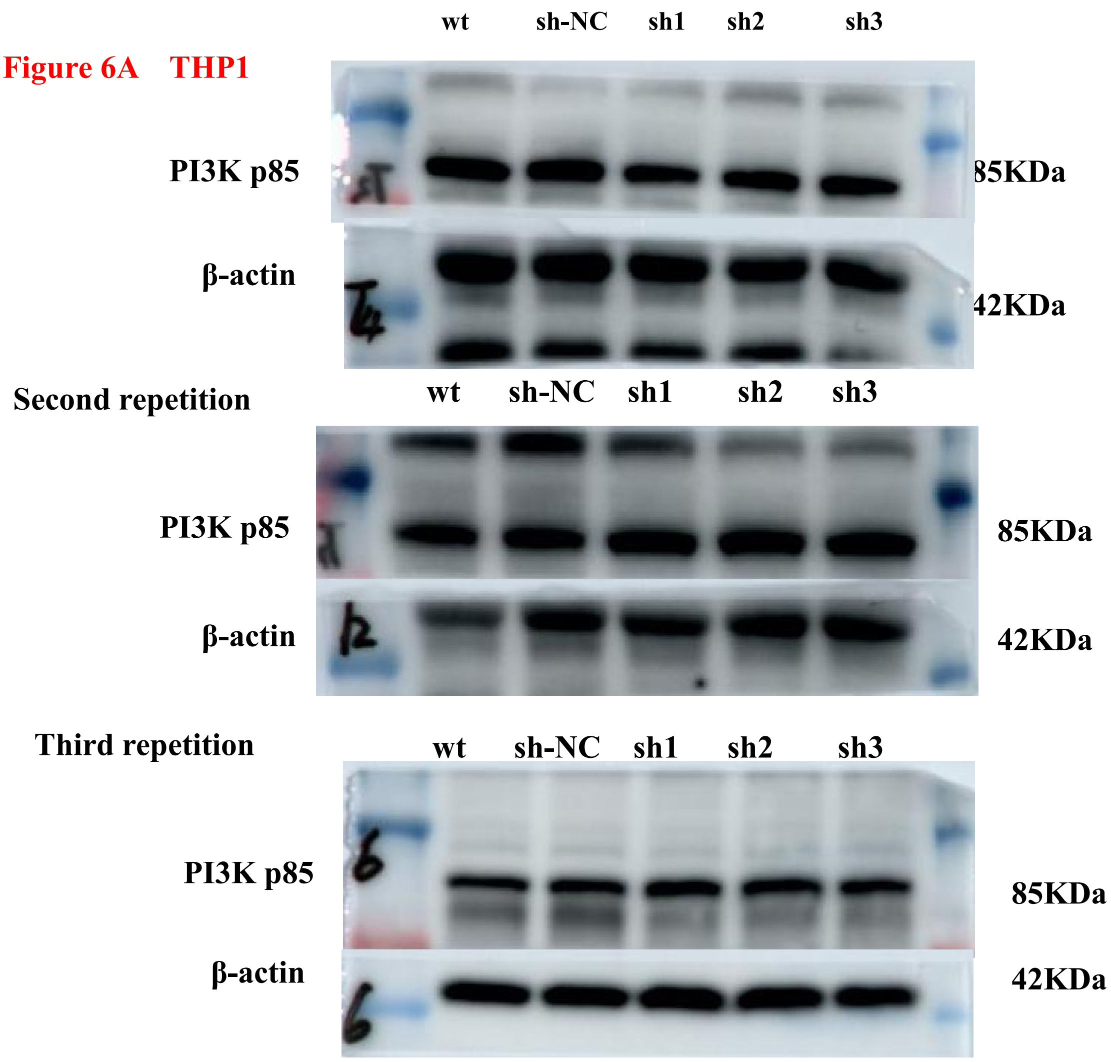

Figure 6A- THP1

wt sh-NC sh1 sh2 sh3

p-PI3K p85

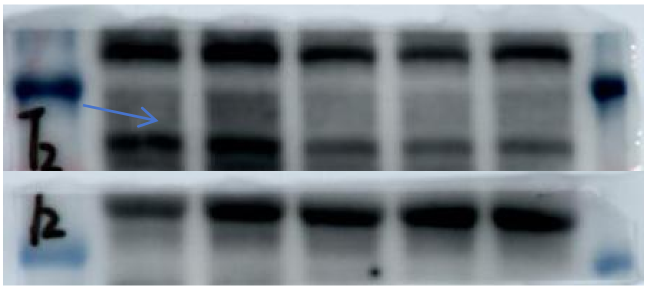

85KDa

β-actin

42KDa

Second repetition

wt sh-NC sh1 sh2 sh3

p-PI3K p85

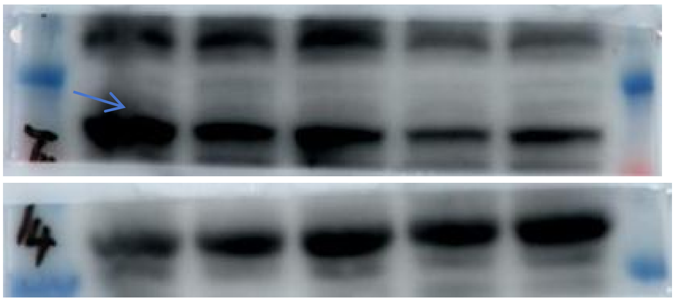

85KDa

β-actin

42KDa

Third repetition

wt sh-NC sh1 sh2 sh3

p-PI3K p85

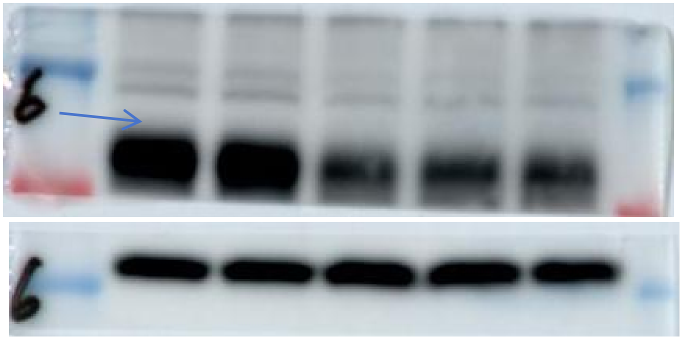

85KDa

β-actin

42KDa

Figure 6A THP1

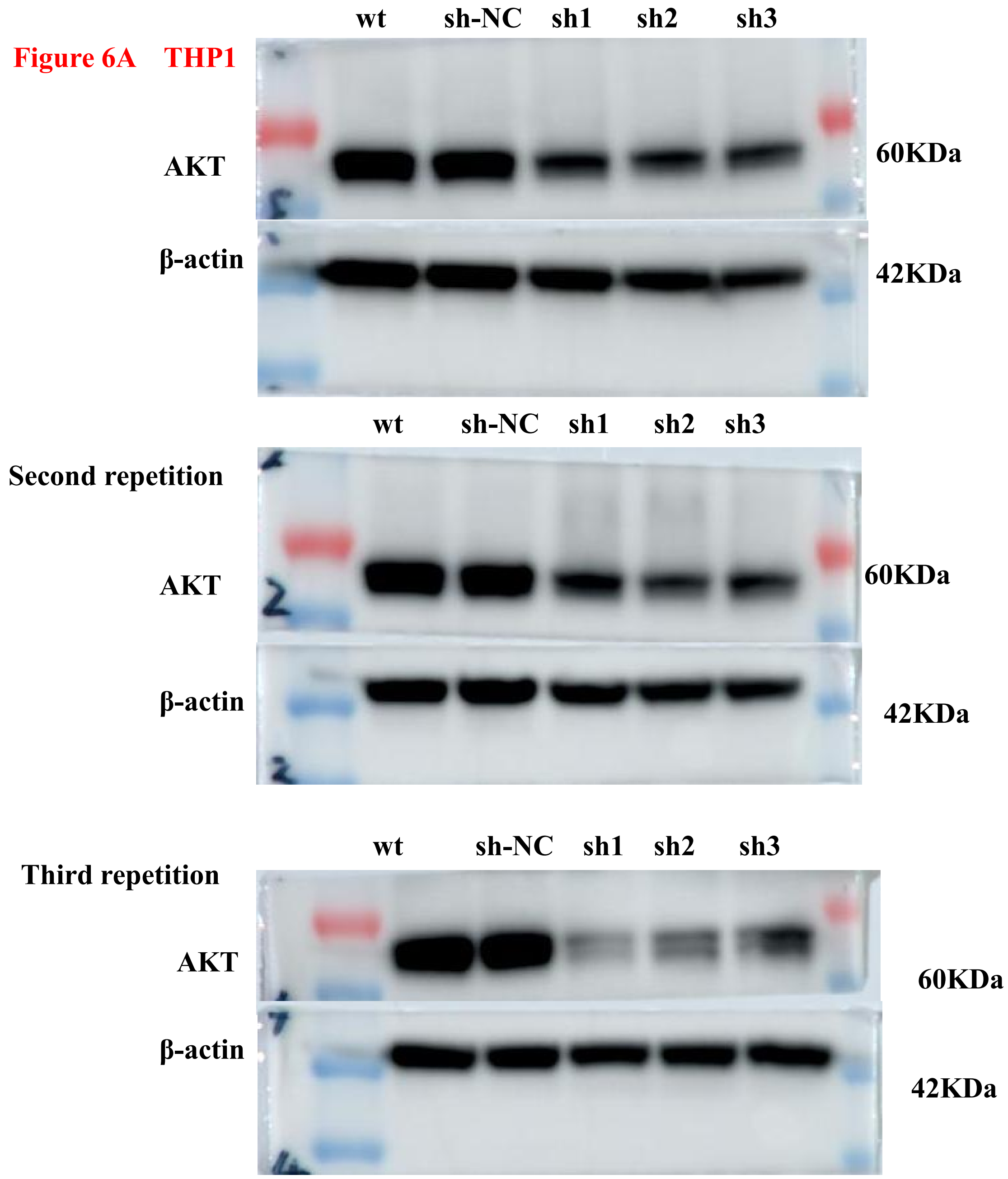

Figure 6A THP1

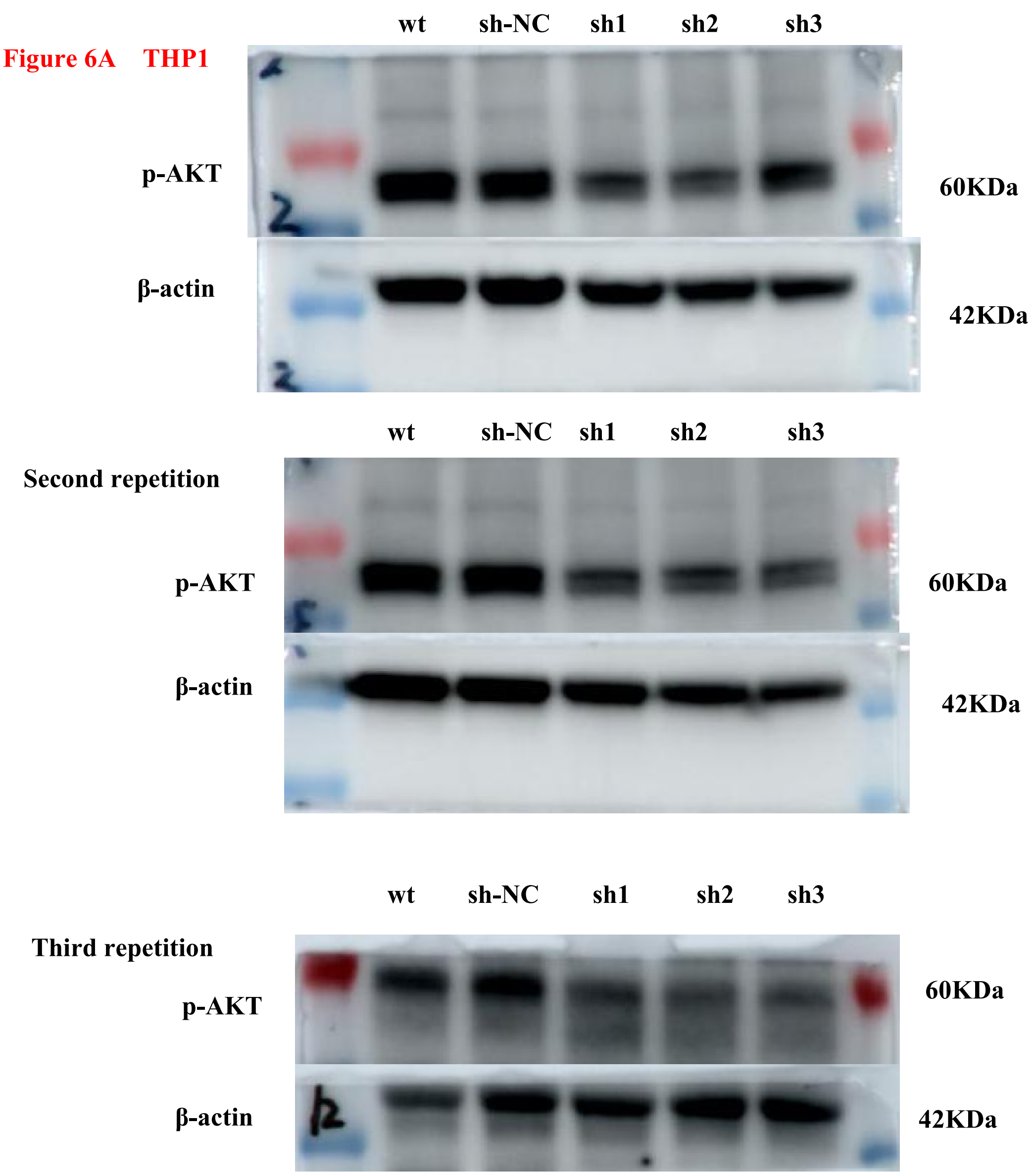

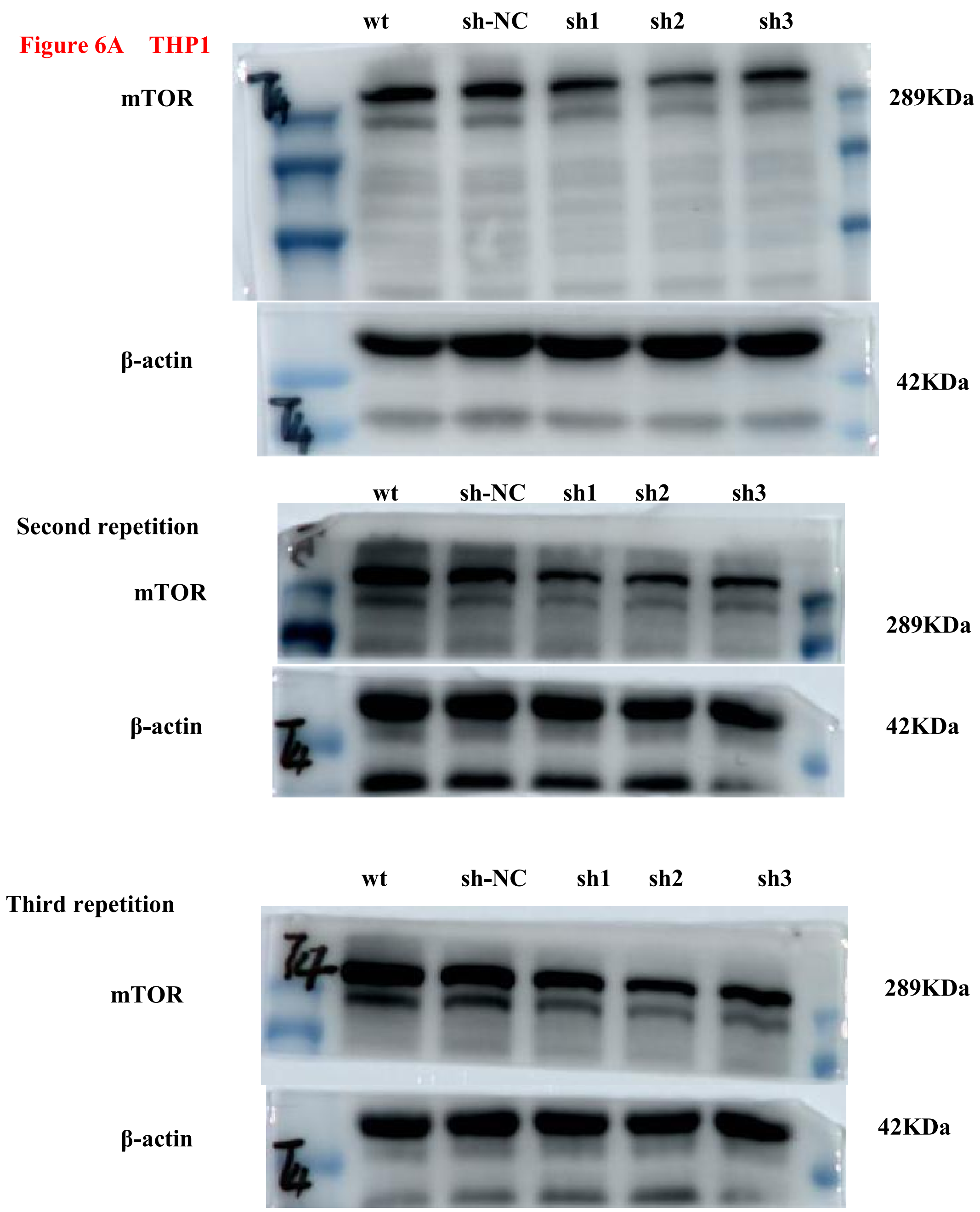
Figure 6A THP1
wt sh-NC sh1 sh2 sh3
mTOR
289KDa
β-actin
42KDa
Second repetition
wt sh-NC sh1 sh2 sh3
mTOR
289KDa
β-actin
42KDa
Third repetition
wt sh-NC sh1 sh2 sh3
mTOR
289KDa
β-actin
42KDa

Figure 6A THP1

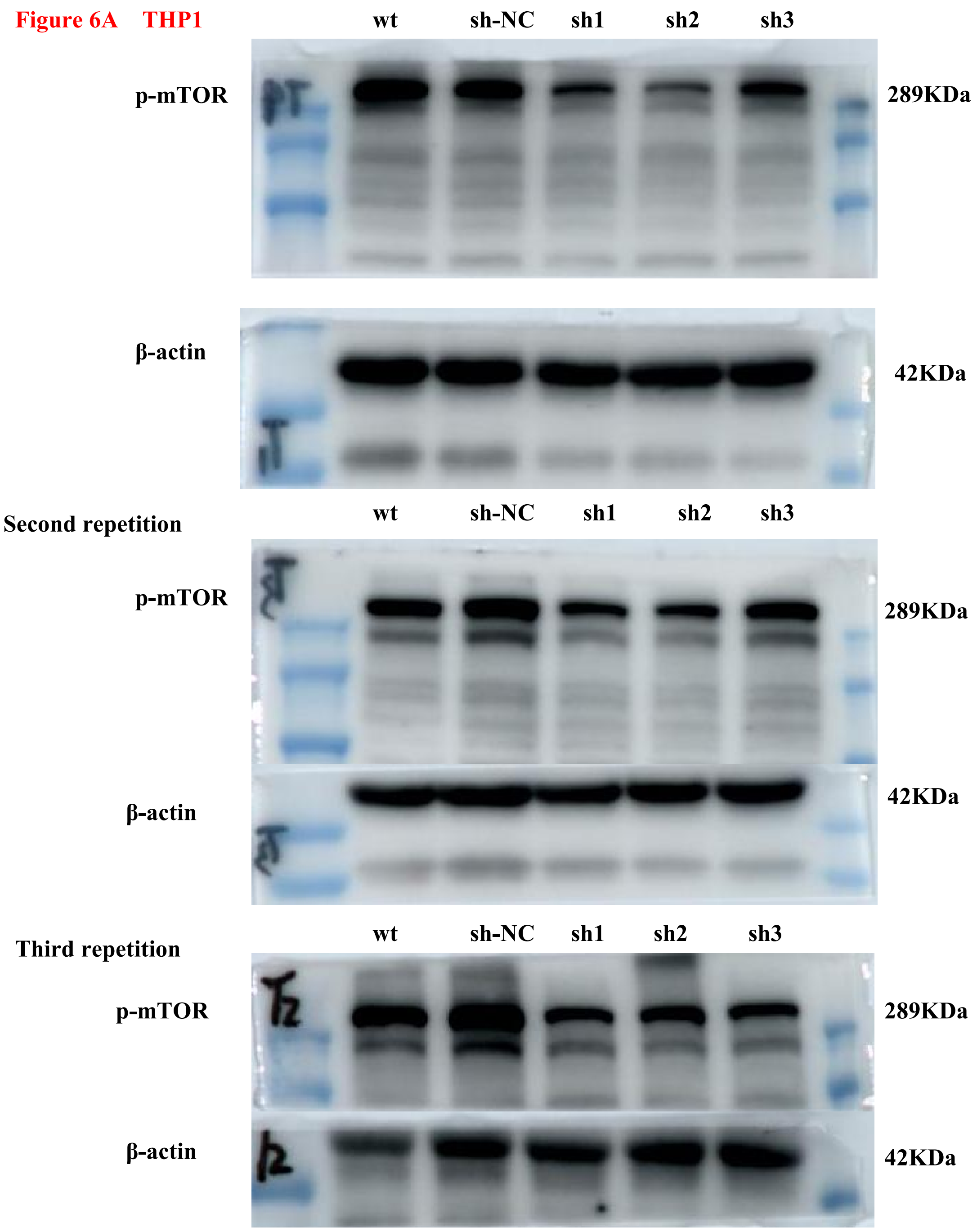

Figure 6B MOLM13

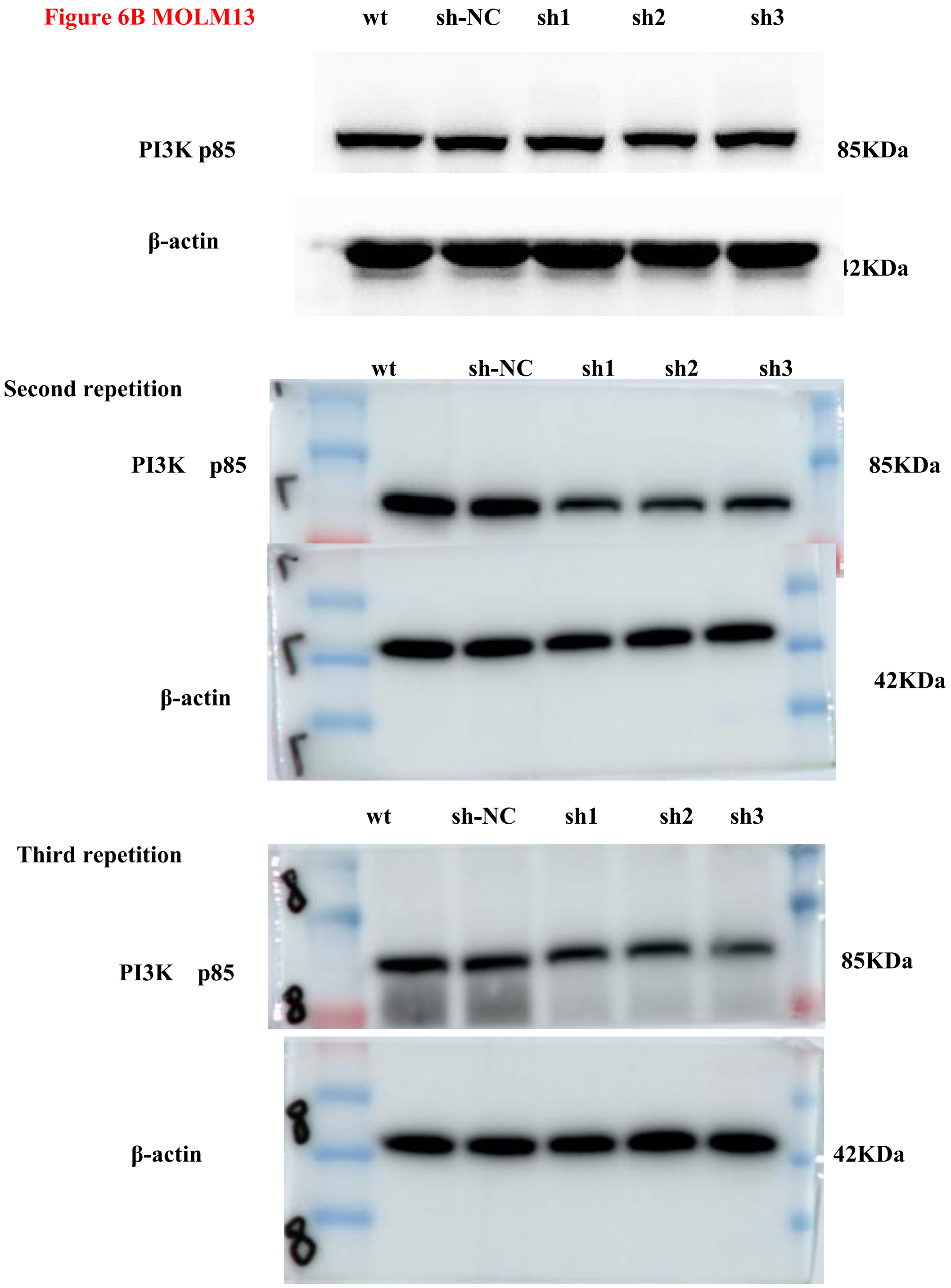

Figure 6B MOLM13

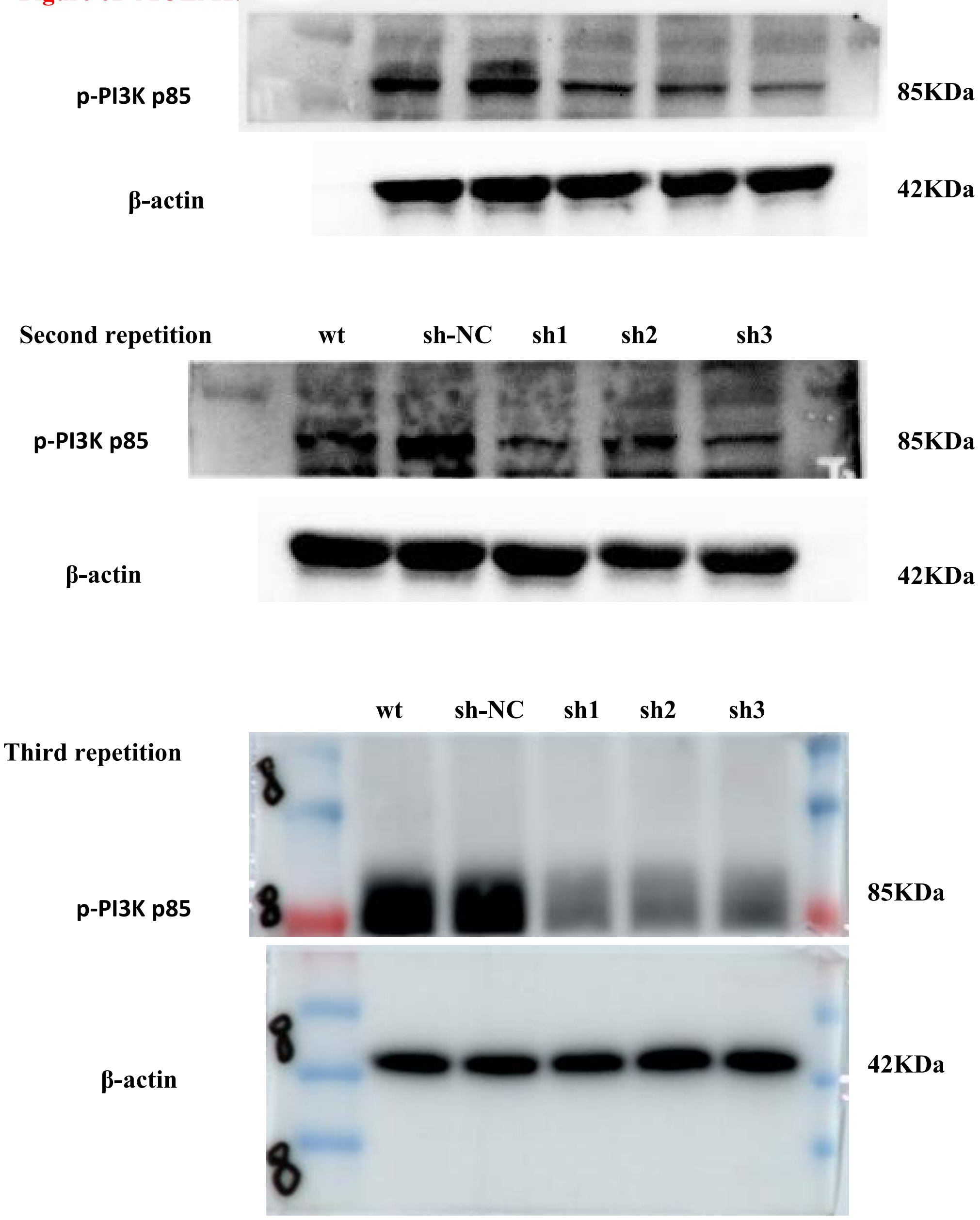

wt sh-NC sh1 sh2 sh3

Figure 6B MOLM13

AKT 60KDa

β-actin 42KDa

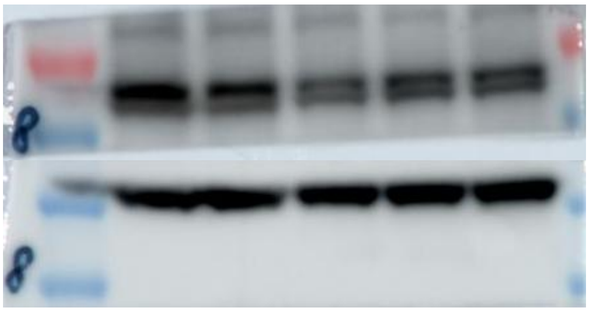

wt sh-NC sh1 sh2 sh3

Second repetition

AKT 150KDa

β-actin 42KDa

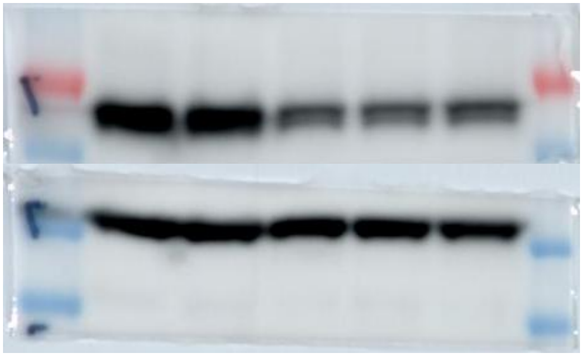

wt sh-NC sh1 sh2 sh3

Third repetition

AKT 42KDa

β-actin 42KDa

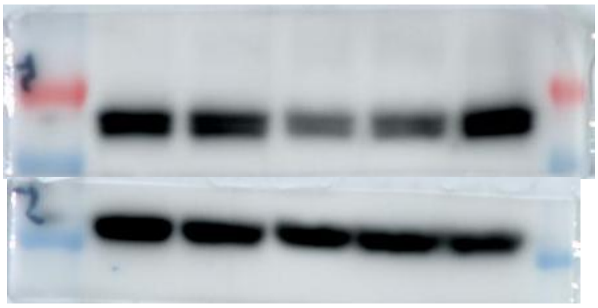

Figure 6B MOLM13

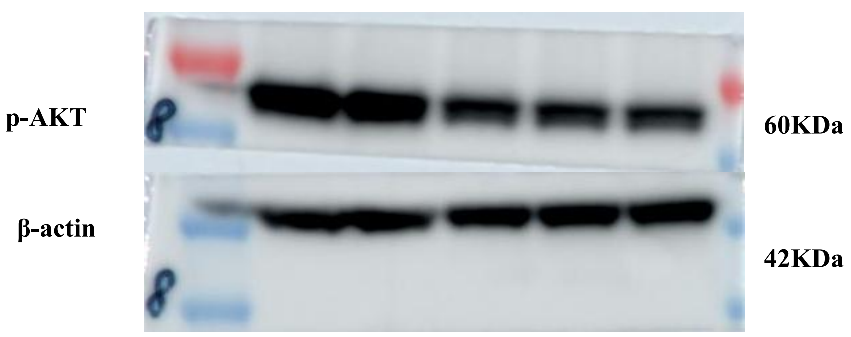

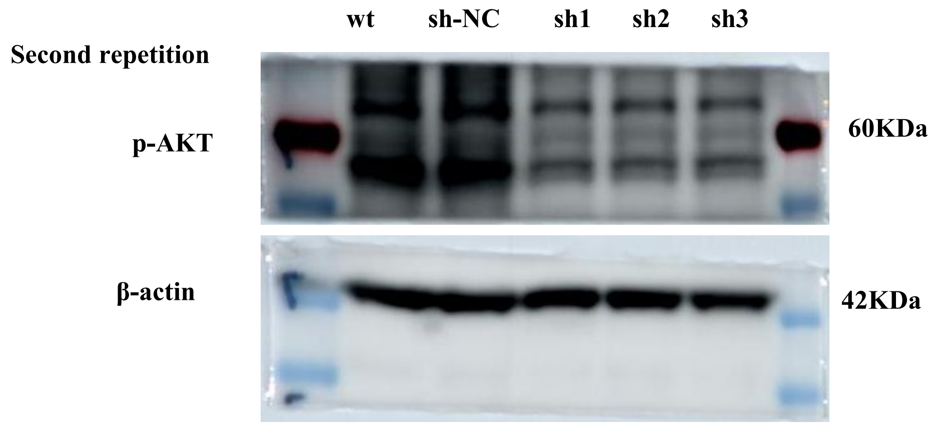

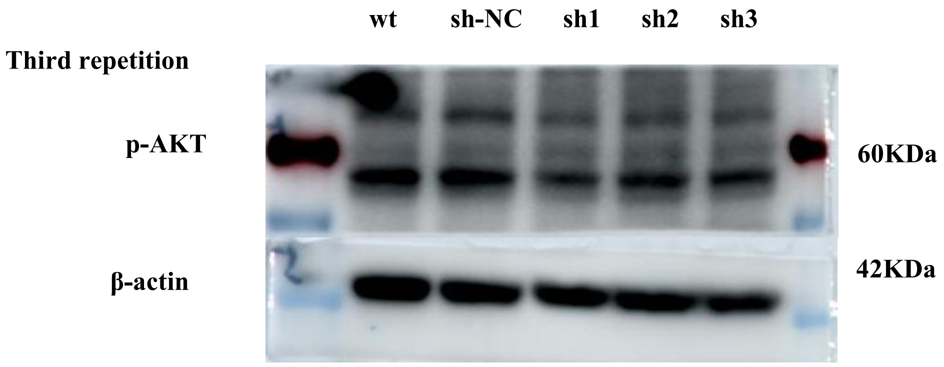

Figure 6B MOLM13

wt sh-NC sh1 sh2 sh3

mTOR 289KDa

β-actin 42KDa

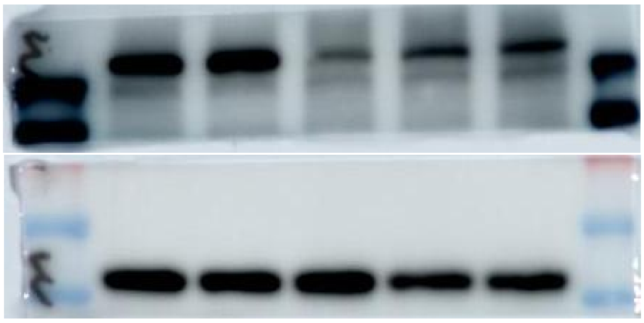

Second repetition

wt sh-NC sh1 sh2 sh3

mTOR 289KDa

β-actin 42KDa

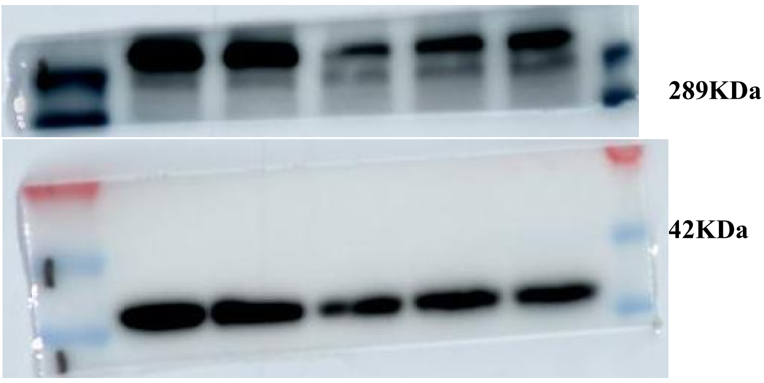

Third repetition

wt sh-NC sh1 sh2 sh3

mTOR 289KDa

β-actin 42KDa

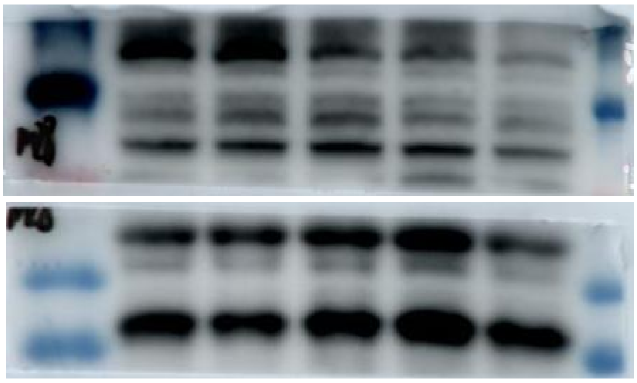

Figure 6B MOLM13

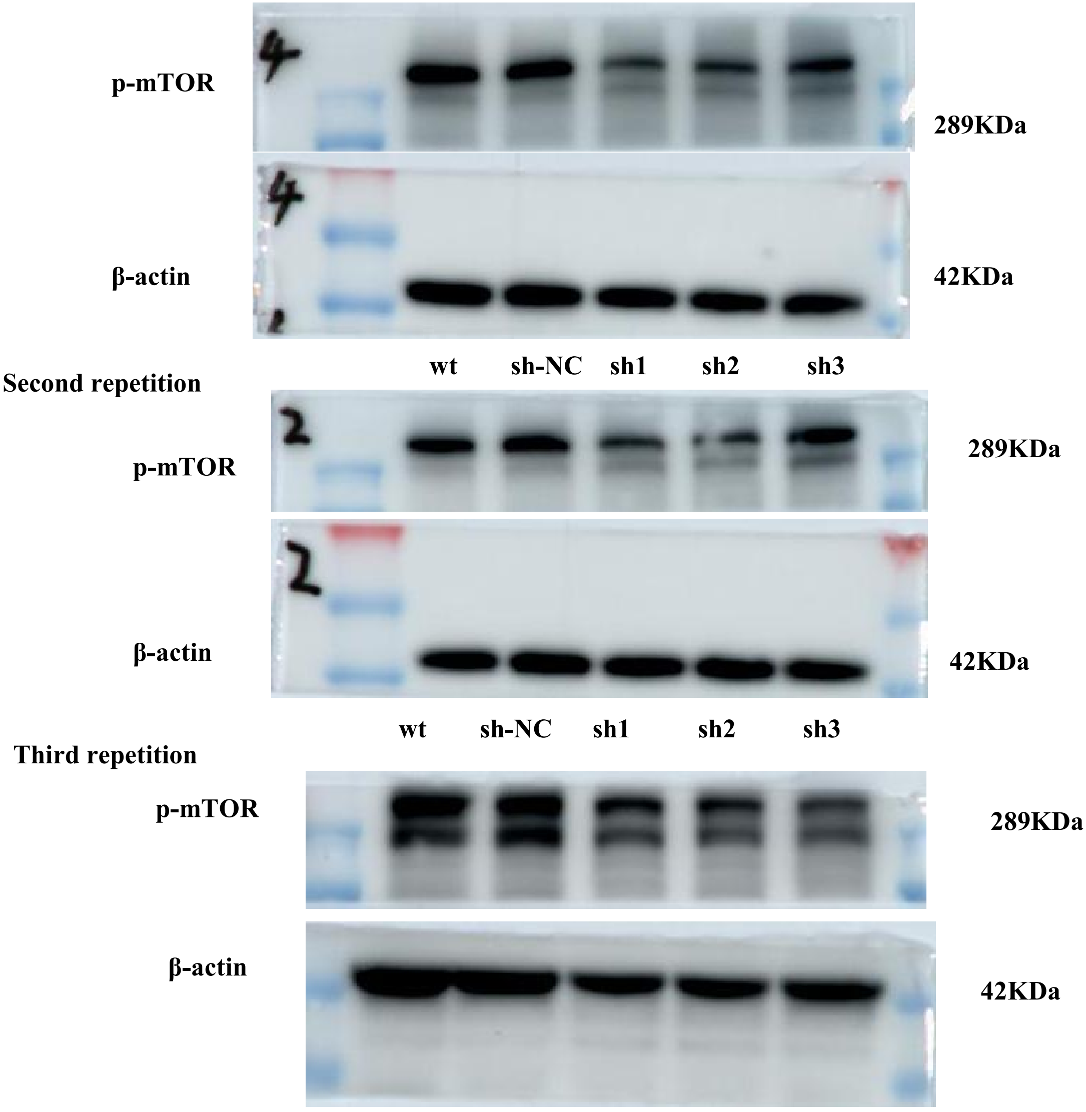

Supplement: Supplementary file 1 [file cancers-17-03076-s001.zip › Supplementary Figure S2 - Original Western Blot images.pdf]
